# ClC-3 regulates the excitability of nociceptive neurons and is involved in inflammatory processes within the spinal sensory pathway

**DOI:** 10.3389/fncel.2022.920075

**Published:** 2022-08-24

**Authors:** Juan Sierra-Marquez, Antje Willuweit, Michael Schöneck, Stefanie Bungert-Plümke, Jana Gehlen, Carina Balduin, Frank Müller, Angelika Lampert, Christoph Fahlke, Raul E. Guzman

**Affiliations:** ^1^Institute of Biological Information Processing, Molecular and Cellular Physiology (IBI-1), Forschungszentrum Jülich, Jülich, Germany; ^2^Medical Imaging Physics, Institute of Neuroscience and Medicine (INM-4), Forschungszentrum Jülich, Jülich, Germany; ^3^Institute of Physiology, RWTH Aachen, Aachen, Germany

**Keywords:** ClC-3, chloride-proton exchanger, neuronal excitability, pain, microglia activation, action potential, DRG

## Abstract

ClC-3 Cl^–^/H^+^ exchangers are expressed in multiple endosomal compartments and likely modify intra-endosomal pH and [Cl^–^] *via* the stoichiometrically coupled exchange of two Cl^–^ ions and one H^+^. We studied pain perception in *Clcn3^–/–^* mice and found that ClC-3 not only modifies the electrical activity of peripheral nociceptors but is also involved in inflammatory processes in the spinal cord. We demonstrate that ClC-3 regulates the number of Na*_*v*_* and K*_*v*_* ion channels in the plasma membrane of dorsal root ganglion (DRG) neurons and that these changes impair the age-dependent decline in excitability of sensory neurons. To distinguish the role of ClC-3 in Cl^–^/H^+^ exchange from its other functions in pain perception, we used mice homozygous for the E281Q ClC-3 point mutation (*Clcn3^E281Q/E281Q^*), which completely eliminates transport activity. Since ClC-3 forms heterodimers with ClC-4, we crossed these animals with *Clcn4*^–/–^ to obtain mice completely lacking in ClC-3-associated endosomal chloride–proton transport. The electrical properties of *Clcn3*^E281Q/E281Q^*/Clcn4^–/–^* DRG neurons were similar to those of wild-type cells, indicating that the age-dependent adjustment of neuronal excitability is independent of ClC-3 transport activity. Both *Clcn3^–/–^* and *Clcn3^E281Q/E281Q^*/*Clcn4*^–/–^ animals exhibited microglial activation in the spinal cord, demonstrating that competent ClC-3 transport is needed to maintain glial cell homeostasis. Our findings illustrate how reduced Cl^–^/H^+^ exchange contributes to inflammatory responses and demonstrate a role for ClC-3 in the homeostatic regulation of neuronal excitability beyond its function in endosomal ion balance.

## Introduction

CLC-type Cl^–^/H^+^ exchangers are expressed in the endoplasmic reticulum, Golgi apparatus, and endosomes/lysosomes, with isoform-specific subcellular distributions (Jentsch and Pusch, 2018; Bose et al., 2021). Their physiological importance has been demonstrated by studies of knockout animal models and by naturally occurring mutations in patients with genetic diseases: genetic ablation or naturally occurring mutations in *CLCN3* or *CLCN7* causes neurodegeneration in the central nervous system (CNS; Kornak et al., 2001; Stobrawa et al., 2001; Dickerson et al., 2002; Kasper et al., 2005; Duncan et al., 2021). Mutations in the *CLCN4* gene are associated with intellectual disability and epilepsy (Veeramah et al., 2013; Hu et al., 2016; Palmer et al., 2018; He et al., 2021b; Guzman et al., 2022), and *CLCN6* mutations result in West syndrome and lysosomal storage disease (Poet et al., 2006; He et al., 2021a). Genetic ablation of ClC-6 or downregulation of ClC-3 alters pain sensitivity in mice (Poet et al., 2006; Pang et al., 2016), suggesting a role for Cl^–^/H^+^ exchangers in pain regulation. However, the functions of CLC transporters in the peripheral nervous system are insufficiently understood.

ClC-3 was suggested to contribute to endosomal acidification (which is initially set by the proton ATPase) by mediating shunting inward chloride currents (Stobrawa et al., 2001). However, the strong outward rectification of ClC-3 (Guzman et al., 2013; Rohrbough et al., 2018) argues against such a function and instead suggests that Cl^–^/H^+^ actively acidifies endosomal compartments by exchanging luminal chloride for protons. However, it is unclear how changes in Cl^–^/H^+^ exchange modify cell function and how the genetic ablation of individual transporters results in neurodegeneration of variable severity. Impaired pain sensitivity might be caused by a variety of cellular processes; therefore, studying the mechanisms underlying hyperalgesia in *Clcn3^–/–^* mice may provide new insight into the cellular functions of CLC-type Cl^–^/H^+^ exchangers.

We found that *Clcn3^–/–^* sensory dorsal root ganglion (DRG) cells are hyperexcitable, likely due to altered plasma membrane densities of Na^+^ and K^+^ channels (Pang et al., 2016). Moreover, we observed microglia activation within the *Clcn3^–/–^* dorsal horn of the spinal cord (DHSC). To distinguish whether these alterations are caused by loss of ClC-3-mediated Cl^–^/H^+^ exchange or ClC-3 chaperone function, we used a knock-in mouse model (*Clcn3^E281Q/E281Q^*) that expresses transport-incompetent ClC-3 E281Q (Guzman et al., 2013). Since ClC-4 is targeted to recycling endosomes and lysosomes in the form of ClC-3/ClC-4 heterodimers, *Clcn3^E281Q/E281Q^*/*Clcn4^–/–^* double-mutant (*DMut*) mice were generated to completely abolish ClC-3-associated Cl^–^/H^+^ exchange. In these double mutants, disruption of endosomal ClC-3-associated Cl^–^/H^+^ exchange did not change the excitability of DRG neurons, suggesting that ClC-3-mediated Cl^–^/H^+^ exchange does not regulate the excitability of nociceptive sensory neurons. However, enhanced microglia activation within the spinal tissue of *Clcn3^–/–^* and *DMut* mice indicates that ClC-3 transport is required to maintain neuroglia homeostasis. Our findings demonstrate how ClC-3 Cl^–^/H^+^ exchangers orchestrate two distinct processes within the sensory pathway.

## Materials and methods

### Animal handling and maintenance

ClC-3 knockout mice (Stobrawa et al., 2001) were kindly provided by Dr. T. Jentsch (Leibniz-Institut für Molekulare Pharmakologie and Max-Delbrück-Centrum für Molekulare Medizin, Berlin, Germany) and maintained in house by breeding heterozygous mice. Heterozygous *Clcn3^+/E281Q^* was generated by Cyagen Biosciences (Santa Clara, CA, United States) by site-directed mutagenesis in the C57BL/6 background. Homozygous animals were obtained by mating heterozygous mice and identified by PCR using the KAPA Mouse Genotyping Kit (Kapa Biosystems/Roche, KK-7302, Wilmington, MA, United States), according to the manufacturer’s instruction. All *Clcn3^–/–^* mice used in this study showed the same phenotype and identical structural changes previously described by others (Stobrawa et al., 2001; Dickerson et al., 2002; Yoshikawa et al., 2002). The lack of commercially available antibodies (Comini et al., 2022) prevented validation of *ClC-3 knockout* at the protein level. *Clcn4^–/–^* mice were generated by Trans Genic Inc. (Kawasaki, Japan), obtained from Deltagen (San Mateo, USA), and maintained as homozygous animals. To generate double-mutant *Clcn3*^E281Q/E281Q^*/Clcn4^–/–^* homozygous mice, homozygous *Clcn4^–/–^* were crossed with homozygous *Clcn3*^E281Q/E281Q^; the subsequent crossing of heterozygous first-generation offspring was used to generate *Clcn3*^E281Q/E281Q^*/Clcn4^–/–^* (Supplementary Figure 1). All animals were genotyped; PCR protocol and primers for genotyping of all mouse modes are provided in Table 1. All mice were housed with a maximum of five mice in the home cage, with food and water ad libitum under controlled conditions with a 12 h–12 h light–dark cycle, air humidity of 55 ± 10%, and a constant room temperature (RT) of 22°C.

**TABLE 1:** List of primers used for genotyping.

Primer	Sequence	Product size (bp)
S for wild type (WT)	GATCTAATTCTGCCTTCCTC	550 WT
S for *Clcn3*^–/–^	GGAAGACAATAGCAGGCATGC	650 Mut
AS for WT/*Clcn3*^–/–^	ACTCTGCCCATGTTTTCCACT	WT/Mut
S for WT	TCTTGCGGCGTGGCCGTCCACCCGG	329 WT
S for *Clcn4*^–/–^	GACGTTGTTTGTCTTCAAGAAGCTTC	628 Mut
AS for WT/*Clcn4*^–/–^	CAAGGGGATGACCGCGAGTGACTGTC	WT/Mut
S for WT/ *Clcn3^E281Q^*	CACGGGATCACAGTAGTGAAAGG	250 WT
AS for WT/ *Clcn3^E281Q^*	CGCTGCAGTCCATTAAACAGTTTC	332 Mut

S, sense primer, or forward primer; AS, antisense primer, or reverse primer.

### Nociceptive testing

Animals of both sexes were used for all nociceptive behavior test experiments, without obvious sex differences. Homozygous female and male mice were compared with their wild-type (WT) littermates at age 21 ± 2 days (P21) or 60 ± 5 days (P60). Before the start of the behavioral test, each mouse was habituated to the testing room for 30 min in a clean single cage. To prevent learning effects, animals were randomly exposed to different temperatures. Each temperature was measured once per day, with at least 1 h between measurements. Animals were rested in empty cages for at least 2 h at RT between the tail flick and hot plate tests.

All behavioral pain experiments were performed by experimenters blinded to the genotype, recorded, and analyzed afterward. For tail flick tests, tails were immersed in water baths (Julabo, Germany) at different temperatures of 46, 48, or 50°C, while the mouse was loosely restrained in the experimenter’s hand. The time before the tail withdrawal was recorded, and each temperature was evaluated in triplicate with a one-day interval between tests. The hot plate test was performed by placing mice onto the metal surface of a heating plate (Ugo Basile S.R.L., Gemonio, Varese, Italy), which was surrounded by a 20-cm-high transparent Plexiglas cylinder and allowed free movement. For this set of experiments, temperatures of 46, 48, 50, and 52°C were used. We avoided higher temperatures, which are often used in this test (Marics et al., 2014), because of the hyperalgesia phenotype of *Clcn3^–/–^*. The video-recorded experiments were carefully analyzed using slow-motion presentations. Since a hyperlocomotion phenotype has been described for *Clcn3^–/–^* mice (Stobrawa et al., 2001), the time period until the animals showed discomfort by licking or shaking the paw was manually analyzed rather than using an automated device. If the animals did not show any reaction within 30 s, they were immediately removed to avoid tissue damage (Marics et al., 2014). The response of each mouse to each temperature was evaluated once per day in triplicate, with 1 h between measurements and one-day interval between tests. After injection of 5 μl 0.5% (v/v) formalin solution into the right hind paw with a Hamilton microliter syringe (Merck KGaA, Darmstadt, Germany), individual mice were placed into a transparent box (19 cm × 19 cm × 11 cm) with three mirrored walls. The number of paw flinches per minute was recorded and analyzed afterward. The number of flinches was manually counted using slow-motion videos. Measurements were repeated every 5 min for a total of 40 min.

### Dorsal root ganglion neuron culture

Dorsal root ganglion neurons were cultured according to a previous protocol with slight modifications (Bost et al., 2017). Dorsal roots were dissected from P21 or P60 animals and cleaned from connective tissue and fibers in ice-cold Locke’s solution (in mM: 154 NaCl, 5.6 KCl, 3.6 NaHCO_3_, 5 HEPES [4-(2-hydroxyethyl)-1-piperazineethanesulfonic acid), 6 glucose, adjusted to pH 7.3 with NaOH]. After treatment with 200 μl TrypLE Express Enzyme (Cat. 12604013, Gibco, Grand Island, NY, United States) for 5 min at RT, ganglia were immediately transferred into a tube containing 1 ml Neurobasal-A medium (Cat. 10888022, Gibco, Grand Island, NY, United States), 20 μl freshly thawed Liberase (DH Research Grade, Cat. 5401054001, Roche Diagnostics Deutschland GmbH, Mannheim, Germany), and collagenase to a final concentration of 2.3 units/ml and incubated in a water bath at 37°C. Neurons were dissociated using a three-step process: a 7-min incubation, followed by pipetting 10 times with a 1-ml pipette; a 5-min incubation, followed by pipetting 10 times; and a final 5-min incubation, followed by pipetting 15–17 times. After adding 200 μl fetal bovine serum (Cat. 10270-106, Gibco, Grand Island, NY, United States) and incubating at 37°C for 3 min with gentle agitation, cells were sedimented at 400 × *g* for 4 min. The cell pellet was then washed with 700 μl Dulbecco’s phosphate-buffered saline (PBS; Cat. 14190094, Gibco, Grand Island, NY, United States). After re-pelleting (400 × *g* for 4 min), freshly prepared NBA-enriched culture medium containing 1% B-27 supplement (Cat. 17504044, Gibco, Grand Island, NY, United States), 1% GlutaMAX (Cat. 35050061, Gibco, Grand Island, NY, United States), 0.4% penicillin/streptomycin (Cat. 15070-063, Gibco, Grand Island, NY, United States), and 5% fetal bovine serum in neurobasal-A medium was added to the cells. The neuronal cell suspension was diluted in 3.5-ml-enriched NBA medium, and approximately, 500 μl cell suspension was seeded onto poly-D-lysine-coated coverslips and cultured at 37°C with 5% CO_2_ and 90% humidity for 1–5 days. At 24 h after plating, a cocktail of uridine and floxuridine thymidylate synthase inhibitors were added to a final concentration of 40 and 100 mM, respectively; 12 h later, the culture medium was replaced with an enriched NBA medium without inhibitors.

### Electrophysiological experiments

Action potentials (APs) were measured using whole-cell recordings in the current-clamp mode under physiological saline solutions, adapted from a published method (Liu et al., 2010; Hoerauf et al., 2015): the bath solution (330 mOsm/kg, adjusted to pH 7.4 with NaOH) contained (in mM) 130 NaCl, 4 KCl, 1 MgCl_2_, 2 CaCl_2_, 10 HEPES, and 48 D-glucose. The pipette solution (310 mOsm/kg, adjusted to pH 7.4 with NaOH) contained (in mM) 135 K-gluconate, 7 NaCl, 2 MgCl_2_, 2 Na-ATP, 0.3 Na_2_-GTP, 10 HEPES, and 0.2 EGTA. The passive and active properties of the cells were obtained from the voltage responses to 1 s rectangular current pulse injections, with a pre- and post-pulse period of 500 ms. Resting membrane potentials (RMPs) were recorded a few minutes after establishing the whole-cell configuration. Neurons were visualized using an Andor’s Neo 5.5 sCMOs camera attached to the microscope, which is regularly calibrated with a calibration slide. Only neurons with a diameter below 23 μm were used for electrophysiology. Only neurons with a stable RMP between –60 mV and –75 mV and with a series resistance of <12 MΩ were included in the analysis. Input resistance (R_in_) was calculated as the slope of the relationship between the voltage response to a current injection within a range from –60 to 20 pA before the first AP. AP thresholds were obtained from the first derivative of the voltage response (Lazarus and Huang, 2011) and AP half-widths from the difference between the rising and decaying phase times at the half-maximum amplitude of the AP. The after-hyperpolarization amplitude (AHP) is defined as the difference between the AP threshold and the minimum voltage response after the peak maximum. All AP properties were analyzed from the first-ever AP using a custom-written Igor-based macro (Igor Pro 7.01 software, WaveMetrics; kindly provided by Dr. Karlijn van Aerde and Dr. Dirk Feldmeyer, INM-10, Forschungszentrum Jülich).

An index to evaluate the intrinsic excitability was used to estimate the excitability of the neuron, as previously described (Lazarus and Huang, 2011): a lower excitability index (EI) indicates a more excitable cell, and a higher EI a less excitable cell (Lazarus and Huang, 2011). The EI is calculated using properties, such as RMP, AP threshold, and R_in_, as follows:

$$E\,I=\left(\frac{A\,P\,t\,h\,r\,e\,s\,h\,o\,l\,d-R\,M\,P}{R_{i\,n}}\right)$$

Sodium and potassium currents were measured using whole-cell patch-clamp recordings in the voltage-clamp mode. To reduce space-clamp errors, healthy DRG neurons with no obvious processes were used within the first 24 h after plating. For sodium currents, the bath solution contained (in mM) 20 NaCl, 105 choline-Cl, 3 KCl, 1 MgCl_2_, 1 CaCl_2_, 10 HEPES, 10 D-glucose, 20 tetraethylammonium chloride, 0.1 CdCl_2_, and 3 4-aminopyridine (4-AP) at 305 mOsm/kg and pH 7.4 (adjusted with choline-OH), and the pipette solution contained (in mM) 7 NaCl, 105 CsF, 10 EGTA, 10 HEPES, and 50 D-glucose at 300 mOsm/kg and pH 7.4 (adjusted with CsOH). To distinguish tetrodotoxin-sensitive (TTX-S) and TTX-resistant (TTX-R) sodium channels, TTX was applied to a final concentration of 300 nM *via* a perfusion pipette, and current recordings were compared before and after the application of TTX (Meents and Lampert, 2016; Fischer et al., 2017). For potassium currents, the bath solution contained (in mM) 150 choline-Cl, 5 KCl, 1 MgCl_2_, 2 CaCl_2_, 10 HEPES, 1 CdCl_2_, and 10 D-glucose at 320 mOsm/kg and pH 7.4 (adjusted with choline-OH), and the pipette solution contained (in mM) 120 K-gluconate, 20 KCl, 2 MgCl_2_, 1 CaCl_2_, 10 EGTA, 10 HEPES, 5 Mg-ATP, and 0.3 Na_2_-GTP at 315 mOsm/kg and pH 7.4 (adjusted with NaOH). To block fast-inactivating potassium currents, 5 mM 4-AP was added to the bath solution. In all cases, the osmolality was adjusted with D-glucose and measured with a freezing point osmometer (Osmomat 3000 basic, Gonotec).

Na^+^ currents were elicited by applying 50-ms test pulses (–100 mV to +40 mV in 5 mV increment every 3 s) from a holding potential of –100 mV. Current inactivation was studied with 500-ms test pulses (–100 mV to +30 mV in 5 mV increment, followed by a 50 ms step at –10 mV) every 3 s from a holding potential of –100 mV. K^+^ currents were elicited by applying 100 ms test pulses (–80 mV to +60 mV in 10 mV increment every 3 s) from a holding potential of –80 mV. Inward-rectifier K channels (IRK) currents were studied during 200-ms test pulses (0 mV to –160 mV in 10 mV decrement every 3 s) from a holding potential of –50 mV.

Recordings were filtered at 10 kHz and sampled at 100 kHz using an EPC10 double patch amplifier, controlled by PatchMaster (HEKA Elektronik). Borosilicate pipettes (GC150F-10, Harvard Apparatus, United States) were pulled with a resistance of 1.9–2.9 MΩ and coated with a thin layer of wax to reduce the capacitance. Cell capacitance (*C*_*m*_) and series resistance (*R*_*s*_) were compensated, and P/4 leak subtraction with a baseline of –80 mV (for K^+^ currents) and −100 mV (for Na^+^ currents) was used to correct linear current components. Peak currents for sodium and mean steady-state currents were plotted against the test voltage to obtain current–voltage relationships. Activation curves for sodium currents were derived by plotting normalized *G*_Na_ as a function of test potential and fitted with the Boltzmann equation (Meents and Lampert, 2016).

### Immunohistochemistry

Dissected spinal cord, dorsal roots, and hippocampus were fixed with 4% PFA for 45 min and retinal tissues for 20 min. All fixed tissues were immersed in a 10% sucrose solution for 1 h at RT and incubated overnight in 30% sucrose at 4°C. After cryoprotection, samples were embedded in optimal cutting temperature compound. Tissue sections (20 μm for the spinal cord, dorsal roots, and retina; 100 μm for the hippocampus) were cut using a cryostat, mounted onto single slides, dried for 5 min at RT, and stored at –20°C until use. To unmask antigens and epitopes (for all antibodies except anti-recoverin and anti-rhodopsin antibodies), sections were treated with sodium citrate buffer (10 mM sodium citrate containing 0.05% Tween 20, pH 6.0 adjusted with NaOH) at 80°C for 15 min and then blocked in PBS containing 0.5% Triton X-100, 10% fetal bovine serum, and 1% bovine serum albumin for 2 h at RT. Samples were incubated with primary antibody (Table 2) diluted in blocking solution for 72 h at 4°C (RT for retinal sections). Slides were then washed five times for 10 min each with 0.1 M phosphate buffer (PB; 100 mM Na_2_HPO_4_ and 100 mM NH_2_PO_4_, adjusted to pH 7.2 with NaOH) and incubated with secondary antibody (Table 2) diluted in blocking solution for 1.5 h at RT in a dark humidified chamber. Finally, slides were washed five times with PB and mounted with Aqua-Poly/Mount on a glass coverslip. Slides were stored overnight at RT until completely dry and stored at –20°C until analysis.

**TABLE 2:** List of antibodies used in immunohistochemistry and Western blot experiments.

Primary antibodies	Catalog N°	Dilution	RRID
Goat-α-Rabbit IgG-Peroxidase, Merk	A6154	1:25000	AB_258284
Anti-mouse α-Tubulin	T9026	1:1000	AB_477593
Anti-rabbit Na_*v*_1.8 (SCN10A), Alomone	ASC-016	1:500	AB_2040188
CGRP Anti-rabbit, Millipore	AB15360	1:4000	AB_672958
NeuN Anti-rabbit, Abcam	AB104225	1:1000	AB_10711153
NeuN Anti-mouse, Millipore	MAB377	1:500	AB_2298772
CD11b Anti-rabbit, Abcam	AB133357	1:500	AB_2650514
Glial fibrillary acidic protein (GFAP) anti chicken, Novus	NB110-58368	1:500	AB_921444
Isolectin GS-IB4 Biotin-XX, Invitrogen	I21414	1:500	AB_2314665
MCP-1 (CCL2) Anti-mouse, Merk	SRP4207	1:500	
TRPV1, Anti-rabbit, Synaptic Systems	444 003	1:1000	AB_2864791
Recoverin, Anti-rabbit, Chemicon	AB5585	1:2000	AB_2253622
Rhodopsin antibody, 1D4, Anti-mouse. R. Molday, University of British Columbia.	MA1-722	1:500	AB_325050 (Thermo Fischer)
**Secondary antibodies and markers**			
Donkey anti mouse Cy3 Dianova	715-165-150	1:200	AB_2340813
Donkey anti rabbit Cy2 Dianova	711-225-152	1:400	AB_2340612
Donkey anti rabbit Cy5 Dianova	711-175-152	1:400	AB_2340607
Donkey anti chicken Cy2 Dianova	703-545-155	1:500	AB_2340375
Streptavidin A488, Thermo Fisher	S11223	1:1000	
TO-PRO™-3 A642, Thermo Fisher	T3605	1:1000	

RRID, Research resource identifier.

### Confocal microscopy and image analysis

Optical images were acquired on a confocal microscope (TCS SP5 II, Leica Microsystems, Germany) using a 20 × /0.70 or 63 × /1.32–0.6 oil immersion objective and digitalized at a resolution of 1024 × 1024 pixels, 200 Hz velocity, and 6-line average in sequential scanning mode. For large tissue imaging, a tile-scan procedure was applied with a 10% stitching threshold. Identical laser intensities and digital gains were used when comparing samples from different phenotypes. Images were processed with FIJI Image J v.1.53c (Wayne Rasband, National Institutes of Health, United States; Schindelin et al., 2012; Rueden et al., 2017), and a self-made pipeline in CellProfiler™ v.3.1.9 cell image analysis software (Broad Institute, Cambridge, MA, United States; Lamprecht et al., 2007; McQuin et al., 2018) was used for automated analysis of cellular fluorescence, area, and shape and number of events. Confocal immunofluorescence images were exported in color and eight-bit grays, and region of interest (ROI) was defined in dorsal horn layers I to IV according to the Allen Brain Atlas of the Mouse Spinal Cord (Lein et al., 2007). Using the point-and-click graphical user interface (GUI), we created a sequential series of modules for image and object processing function. For neuronal counting, “Neurons” for the NeuN staining and “nuclei” for the To-Pro-3 were applied using a global or adaptive threshold strategy. Both objects were delimited using OTSU image processing with either two- or three-class thresholding with a threshold correction factor of 1.0 and a minimum lower bound of 0.2 (pixels below this number were not considered). Neurons and nuclei were identified using a shape method that used peak brightness to identify like-rounded objects. The same approach was used to identify the processes of the astrocytes, microglia, and chemokine ligand (CCL2) staining. The percentage of the area covered by each channel was obtained by dividing it by the area of the ROI. This protocol was standardized with pictures from all phenotypes, and then a high-throughput analysis was done for the whole data set. Although the pipeline was helpful for quantification in the dorsal horn of the spinal cord, counting the DRG neurons was only possible manually using ImageJ.

### Western blotting

Tissue samples from at least two animals were homogenized in ice-cold homogenizing buffer containing 2 mM EDTA pH 8.0, 1 mM cOmplete™ (Cat. 5056489001, COEDTAF-RO, Roche, Mannheim, Germany), and protease inhibitor cocktail mPIC, 1:500 dilution (Cat. P8340, Sigma Aldrich- Merck KGaA, Darmstadt, Germany) and sedimented at 100,000 × *g* for 15 min at 4°C. Pellets were resuspended in solubilization buffer (1% SDS, 10 mM NaPOi (phosphate buffer composed of 92.2 mmol Na_2_HPO_4_ and 6.8 mmol NaH_2_PO_4_), and 1 mM cOmplete™) and sedimented at 15000 × *g* for 10 min. Samples were heated at 95°C for 5 min with a 4× loading buffer (200 mM Tris-Cl, pH 6.8, 8% SDS, 4% 2-mercaptoethanol, 50% glycerin, and 0.04% bromophenol blue), and proteins were resolved by polyacrylamide gel electrophoresis. After transfer to PVDF membrane using the semi-dry transfer technique, membranes were dried overnight, blocked for 2 h in 5% nonfat milk solution in PBS containing 0.05% Tween-20 (PBS-T) or in PBS-T containing 3% bovine serum albumin, and incubated overnight at 4°C with primary antibody (anti-rabbit Na_*v*_1.8 sodium channel, or mouse anti-α-tubulin; Table 2). Membranes were washed with PBS-T and incubated for 1 h with goat-α-rabbit IgG-horseradish peroxidase (HRP) secondary antibody (Table 2). Membranes were exposed to an equal amount of enhanced chemiluminescent Super Signal™ West Pico PLUS (Cat. 34579, Thermo Scientific, Waltham, MA, United States) substrate, and the exposure time was adjusted depending on protein abundance (1–10 min) to maximize band visibility and minimize background.

### Real-time PCR

Total RNA was extracted from freshly dissected DRG neurons from two-month-old mice using the TRIzol™ (Cat. 15596026, Invitrogen, Carlsbad, CA, United States)-chloroform method, and 1 μg total RNA was converted into cDNA using One-Step SuperScript™ III^®^ reverse transcriptase (Cat. 12574018, Invitrogen, Carlsbad, CA, United States) in a Gradient Thermocycler (Biometra, Göttingen, Germany). The master mix contained 12.5 μl 2× reaction mix (containing 0.4 mM each dNTP and 3.2 mM MgSO_4_), 1 μl enzyme, 0.5 μl 10 mM sense/antisense primers (specific for each isoform), 1 μg template RNA, and RNA-free water to a final volume of 25 μl. cDNA was synthesized by incubation for 30 min at 55°C, followed by 2 min at 94°C. PCR conditions were 40 cycles of 15 s at 94°C (denaturation), 30 s at 55–65°C (annealing), and 5 min at 68°C for the extension, with a final step of 1 min at 68°C. The reference gene was 18S RNA. Primers to identify the ClC isoforms are listed in Table 3. PCR products were assessed by 1% agarose gel electrophoresis [with SYBR™ safe (Cat. S33102, Invitrogen, Carlsbad, CA, United States) staining] at 120 V for 35 min. PCR band size was determined using a GeneRuler 100 bp DNA Ladder (Cat. SM0322, Thermo Scientific, Waltham, MA, United States).

**TABLE 3:** List of primers used to identify the ClC exchangers in dorsal root ganglion (DRG).

Isoform	Sequence	Type	Size (bp)
ClC-3a	5′-CGCCCAGCTTGCTATGCCTCTGAG-3′	Forward	324
	5′-AGCTAGTGCCCCTGATGCCAGTC-3′	Reverse	
ClC-3b	5′-CGCCCAGCTTGCTATGCCTCTGAG-3′	Forward	500
	5′-AGCTAGTGCCCCTGATGCCAGTC-3′	Reverse	
ClC-3c	5′-ATGGATGCTTCTTCTGATCC-3′	Forward	379
	5′-AGCTAGTGCCCCTGATGCCAGTC-3′	Reverse	
ClC-4	5′-GACGTGGGGACCTACGAGGACTTCC-3′	Forward	508
	5′-CACTCAAAATAGTCTTTATCTCGGGTATGCC-3′	Reverse	
18S	5′-CAGTATGACTCCACTCACGGCAAATTC-3′	Forward	530
	5′-CACAGTCTTCTGGGTGGCAGTGATG-3′	Reverse	

### Data analysis

Statistical analysis was performed using OriginPro version 2018b (OriginLab Corporation, Northampton, MA, United States) and Microsoft Excel (Microsoft 365). Data are presented as the mean ± standard error of the mean (SEM) and plotted as boxplots (with boxes indicating the upper and the lower quartiles and whiskers the upper and lower 90 percentiles). The non-paired two-tailed *t*-test or the Mann–Whitney test was used to compare two groups, depending on whether the data followed a normal distribution. For comparing more than two groups, one-way ANOVA was used for normally distributed data without significant variance inhomogeneity. For more than two factors or independent variables, and one dependent variable, two- or three-way ANOVA was used. If *F*-value was statistically significant (*p* < 0.05), a Tukey *post hoc* test was conducted.

Each data sample was collected randomly and considered independent. We used the Shapiro–Wilk test to evaluate if the data followed a normal distribution and Levene’s test for assessing the homogeneity of variances. Data that did not follow a normal distribution were normalized. If normalization was not possible or variances were not homogenous, a non-parametric test (Mann–Whitney or Kruskal–Wallis ANOVA test) was chosen. Grubb’s test was used to test for outliers. The *n* value provides the number of cells (or measurement), and N, the number of animals; both values are given in the figure legends. Differences with *p*-values < 0.05 were considered significant. Asterisk (*) was used to represent differences between groups and (‡) when also animals were compared (^*‡^*p* < 0.05, ^**^‡‡*p* < 0.01; ^***^‡‡‡*p* < 0.001).

## Results

### Genetic ablation of ClC-3 leads to hyperalgesia

**FIGURE 1:**
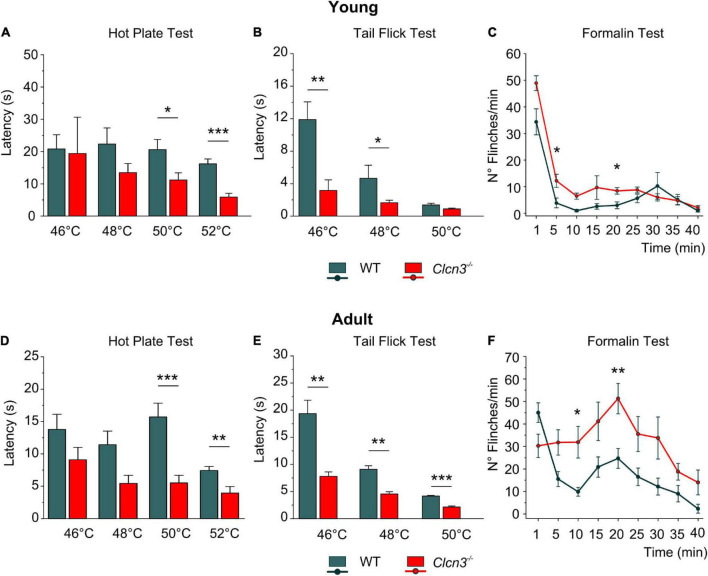
Acute pain experiments in young P21 **(A–C)** and adult P60 **(D–F)** mice show increased thermal sensitivity and lower latencies in mutants compared with wild type (WT) animals. **(A)** Hot plate test, P21; WT (*n* = 7); *Clcn3*^–/–^ (*n* = 8). **(B)** Tail flick test, P21; WT (*n* = 8); *Clcn3*^–/–^ (*n* = 9). **(C)** Formalin test, P21; WT (*n* = 8); *Clcn3*^–/–^ (*n* = 9). **(D)** Hot plate test at different temperatures, P60; WT (*n* = 11); *Clcn3*^–/–^ (*n* = 13). **(E)** Tail flick test, P60; WT (*n* = 8); *Clcn3*^–/–^ (*n* = 9). **(F)** Formalin test, P60; WT (*n* = 6); *Clcn3*^–/–^ (*n* = 8). Statistical significance levels are *p < 0.05, **p < 0.01, ***p < 0.001. *Clcn3*^–/–^ versus WT; *n* represents the number of animals. Two-way ANOVA or three-way ANOVA analyses were used to detect interactions between variables, and Tukey *post hoc* test for pairwise comparisons. Data are presented as the mean ± SEM.

*Clcn3^–/–^* mice suffer from neurodegeneration in the hippocampus and the retina, with loss of neurons beginning at P21 and completed at P60 (Stobrawa et al., 2001). Since neurodegeneration might affect pain sensitivity, we evaluated pain sensitivity before and after neuronal loss at P21 (young) and P60 (adult). At both developmental stages, we found significantly shorter paw withdrawal latencies to heat stimuli in *Clcn3^–/–^* mice than in WT mice (Figures 1A,D). The tail flick test confirmed that *Clcn3^–/–^* mice are more sensitive to noxious thermal stimuli than the WT animals (Figures 1B,E). This phenotype was consistently observed for all temperatures evaluated (Supplementary Table 1). In contrast to our results, a previous report did not identify differences in thermal pain perception between *Clcn3^–/–^* and WT mice (Pang et al., 2016). Pang et al. (2016) used an automated hot plate device that measured the latency of paw licking, rearing, and jumping in triplicate at 15-min intervals. Repeated pain exposure may have modified reaction latencies in these experiments (Suaudeau et al., 2005) and may have masked the differences in response to noxious temperatures we observed between WT and *Clcn3*^–/–^ (Figure 1). We, therefore, measured the effects of each temperature only once per day with at least 1 h between measurements.

Subcutaneous injections of formalin (0.5%) into the hind paw trigger a biphasic pain reaction, comprising an early phase (1–5 min post-injection; Figures 1C,F) due to direct activation of peripheral nociceptors and an inflammatory late-phase reaction (10 min post-injection) associated with persistent pain signals at the supraspinal level (Taylor et al., 1995; Abbadie et al., 1997). Young *Clcn3*^–/–^ were more sensitive to noxious stimuli at both phases, with significantly more flinches per min in the mutant than in WT animals (Figures 1C; Supplementary Table 2). However, in the late inflammatory phase, only adult *Clcn3*^–/–^ mice showed more severe reactions than WT (Figure 1F; Supplementary Table 2), suggesting that lack of ClC-3 alters behavioral pain perception at all ages.

### Ablation of ClC-3 changes the excitability of sensory dorsal root ganglion neurons

**FIGURE 2:**
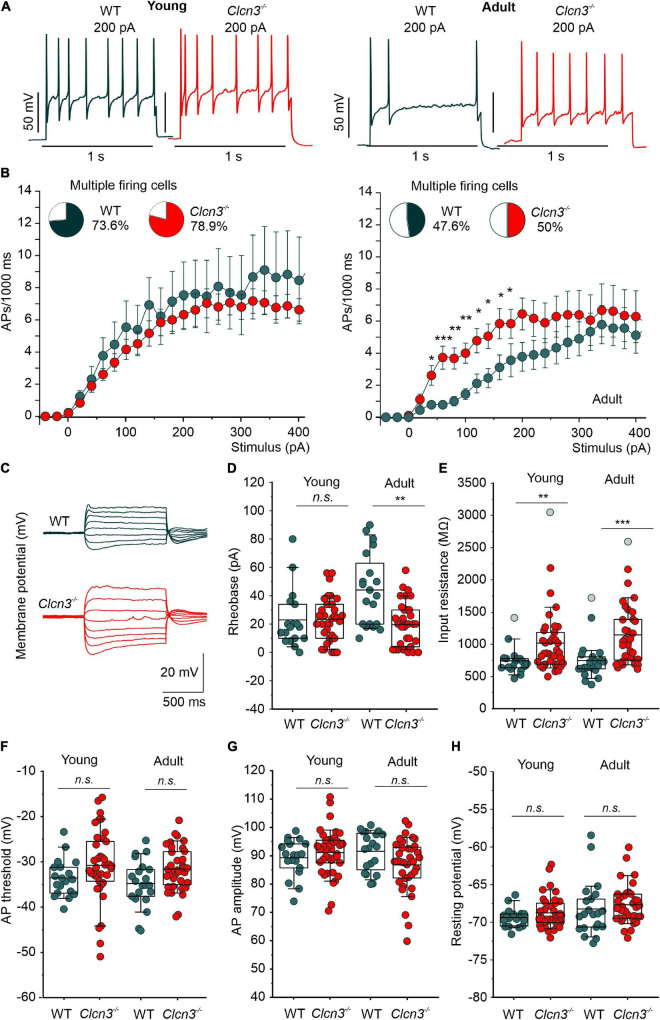
Change in excitability of dorsal root ganglion (DRG) neurons from young and adult *Clcn3*^–/–^ animals. **(A)** Representative traces of action potentials (APs) elicited by a current injection of 200 pA in young (left) and adult (right) wild type (WT) (green) and *Clcn3*^–/–^ (red) neurons. **(B)** Average firing frequencies from young and adult *Clcn3*^–/–^ and WT dorsal root ganglion (DRG) neurons. Insets show the percentage of multiple firing neurons for each condition. **(C)** Representative current responses of control (green) and a *Clcn3*^–/–^ (red) neuron to hyperpolarizing and depolarizing current pulses. **(D)** Current threshold necessary to elicit the first action potential (AP; rheobase) in young and adult *Clcn3*^–/–^ and WT DRG neurons. **(E)** Average input resistances of DRG neurons from P21 and P60 mice. Symbols in gray are outliers values included in the analysis. **(F)** AP thresholds, **(G)** amplitudes, and **(H)** RMP for young and adult neurons. Data were collected from young WT (*n* = 19 cells from four animals) and *Clcn3*^–/–^ (*n* = 38 cells from four animals) and adult WT (*n* = 21 cells from seven animals) and *Clcn3*^–/–^ (*n* = 36 cells from six animals) cells. Statistical significance levels are *p < 0.05, **p < 0.01, ***p < 0.001, n.s., not significant. A two-way ANOVA or Kruskal–Wallis ANOVA was used to compare the age and phenotype of the two groups. The Tukey *post hoc* test was used for pairwise comparisons. Data are presented as the mean ± SEM. In boxplots, boxes indicate the upper and lower quartiles, and whiskers the upper and lower 90 percentiles.

We next examined the electrical properties of sensory neurons from young and adult WT and *Clcn3*^–/–^ mice using current-clamp recordings. Similar numbers of APs were recorded in DRG neurons from young WT and *Clcn3*^–/–^ upon injection of depolarizing currents (Figures 2A,B). However, although the firing rates decreased in older WT mice, this developmental change was not observed in mutant mice (Figures 2A,B). The rheobase current (i.e., the minimum current amplitude required to elicit an AP) was reduced in *Clcn3*^–/–^ adult neurons (Figure 2D), with membrane input resistances significantly higher in both young and adult *Clcn3*^–/–^ animals (Figures 2C,E). AP thresholds and after-hyperpolarization amplitudes (AHP) were slightly shifted toward more depolarizing potentials in *Clcn3^–/–^* mice compared with WT mice (Figure 2F; Supplementary Figure 2), but AP amplitudes and RMPs were unchanged (Figures 2G,H). We also compared the first, second, third, and fourth APs for WT and mutant adult cells and found that AP thresholds, amplitudes, and AHP were not different (Table 4).

**TABLE 4:** Properties of the action potentials (APs) generated in response to 200 pA current injection for 1 s were not different between wild type (WT) and *ClCn3^–/–^* adult dorsal root ganglion (DRG) neurons.

200 pA of current injection	Phenotype	V. Threshold (mV)	Amplitude (mV)	AHP (mV)
*First Action potential* (AP)	WT (*n* = 9)	–59.0 ± 4.7	118.1 ± 5.4	–11.8 ± 3.9
	***Clcn3*^–/–^ (*n* = 16)**	–62.6 ± 2.1	123.6 ± 2.6	–16.4 ± 2.4
*Second AP*	WT (*n* = 9)	–17.0 ± 2.5	69.8 ± 4.3	27.0 ± 2.1
	***Clcn3*^–/–^ (*n* = 16)**	–15.1 ± 1.3	66.8 ± 3.0	28.9 ± 1.7
*Third AP*	WT (*n* = 9)	–16.3 ± 2.2	67.8 ± 4.2	25.5 ± 1.9
	***Clcn3*^–/–^ (*n* = 16)**	–14.0 ± 1.3	64.3 ± 3.3	28.9 ± 1.6
*Fourth AP*	WT (*n* = 4)	–11.7 ± 3.8	57.8 ± 6.6	26.6 ± 3.9
	***Clcn3*^–/–^ (*n* = 16)**	–12.3 ± 1.4	61.0 ± 3.9	29.4 ± 1.7

No statistical differences were found. Significance levels p > 0.05 using Student’s t-test. Data are presented as mean ± SEM.

We next compared the EI for WT and *Clcn3^–/–^* cells. The EI integrates multiple cellular properties (see the equation in the “Materials and methods” section), such as RMP, AP threshold, and the input resistance (R_in_) to estimate the intrinsic excitability of a cell (Lazarus and Huang, 2011), with a lower EI indicating increased excitability. Consistent with the age-dependent reduction in neuronal excitability, we obtained lower EIs for DRG neurons from young WT mice compared with adults. However, in DRG neurons from *Clcn3*^–/–^ mice, EI values did not change with age, resulting in significant differences between adult WT and mutant cells (young DRG, WT 58 ± 4.0 pA vs. *Clcn3*^–/–^ 47 ± 3.0 pA, Student’s *t*-test *p* = 0.05; adult DRG, WT 74 ± 9.0 pA vs. *Clcn3*^–/–^, 42 ± 3.0 pA, Mann–Whitney test *p* = 0.0017). We conclude that ClC-3 transporters contribute to the age-dependent regulation of neuronal excitability in sensory DRG cells.

### *Clcn3* deletion alters ion channel density in the plasma membrane of dorsal root ganglion neurons

**FIGURE 3:**
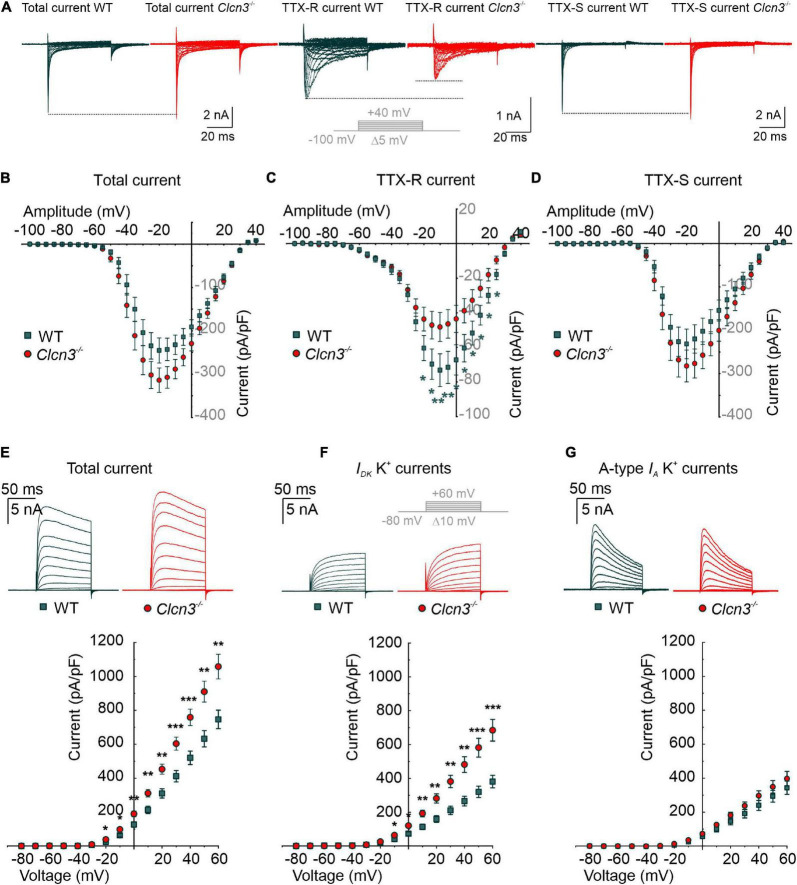
Whole-cell Na^+^ and K^+^ currents from cultured wild type (WT) and *Clcn3*^–/–^ dorsal root ganglion (DRG) adult neurons. **(A)** Representative traces of Na^+^ currents before (total Na^+^ current) and during the application of 300 nM TTX (TTX-R current), and after digital subtraction of the TTX-R current from the total Na^+^ current (TTX-S current) from WT (green) and *Clcn3*^–/–^ (red) cells. The inset illustrates the voltage protocol used to elicit Na^+^ currents during and after TTX application. **(B–D)** Current–voltage relationships of total **(B)**, TTX-R **(C)**, and TTX-S **(D)** Na^+^ current densities from WT (green, *n* = 23 from five animals) and *Clcn3*^–/–^ (red, *n* = 23 from eight animals) DRG neurons. Traces were aligned to the holding current at –100 mV, and dotted lines were set to the maximum pick current of the WT to illustrate changes in the *Clcn3*^–/–^. **(E–G)** Representative recordings of the K^+^ current (upper panel) and current–voltage relationship (lower panel) were obtained from WT (green, *n* = 16 from five animals) and *Clcn3*^–/–^ (red, *n* = 20 from three animals) DRG neurons. Inset in gray depicts the voltage protocol used to elicit K^+^ currents during and after 4-AP application. **(E)** Total K^+^ current densities are significantly higher in the absence of ClC-3. **(F)** Slow-inactivating K^+^ currents obtained from the same neuron showed in **E**, after the application of 5 mM 4-AP, a compound that blocks fast-inactivating K^+^ channels, showed a similar phenotype as for the total K^+^ current. **(G)** Fast-inactivating K^+^ currents are obtained by subtracting the slow-inactivating (**F**) from the total **(E)** current. Statistical significance levels are *p < 0.05, **p < 0.01, and ***p < 0.001; Student’s *t-*test. Data are presented as mean ± SEM.

Voltage-gated sodium channels are necessary to initiate and propagate APs, and changes in the expression or function of these channels are associated with various pain disorders (Cummins and Waxman, 1997; Berta et al., 2008; Dib-Hajj et al., 2010; Lampert et al., 2010; Bernal, 2018). Small-diameter DRG neurons express fast-inactivating TTX-sensitive (TTX-S) Na_*v*_1.1, Na_*v*_1.6, and Na_*v*_1.7 and slow-inactivating TTX-resistant (TTX-R) Na_*v*_1.8 and Na_*v*_1.9 (Rush et al., 2007) channels. In patch-clamp experiments, we measured sodium currents in DRG neurons from *Clcn3^–/–^* and WT adults before and during the extracellular application of 300 nM TTX, using the same voltage protocol. Total Na^+^ current densities were slightly increased in *Clcn3^–/–^* DRG neurons (Figures 3A,B), along with a significantly decreased TTX-R component (Figures 3A,C). To determine the TTX-S Na^+^ current, the TTX-R component was subtracted from the total Na^+^ current. The TTX-S current did not differ between *Clcn3^–/–^* and WT cells (Figures 3A,D). Western blotting showed that Na_*v*_1.8 protein levels were not reduced in *Clcn3^–/–^* cells (Supplementary Figure 3), indicating that loss of ClC-3 does not affect the *de novo* synthesis of Na_*v*_1.8 channels. We observed minor changes in the voltage dependence of sodium channel activation and inactivation in *Clcn3^–/–^* neurons compared with WT neurons (Supplementary Figures 4A–F), probably due to changes in the relative amplitudes of different sodium channels in DRG neurons. Taken together, these observations suggest that ClC-3 regulates the surface membrane insertion of the TTX-R Na_*v*_1.8/Na_*v*_1.9, but not of the Na_*v*_1.1, Na_*v*_1.6, or Na_v_1.7 channels.

Altered nociception has also been associated with changes in the surface expression and biophysical properties of K^+^ channels (Duan et al., 2012; Laumet et al., 2015; Conner et al., 2016). In whole-cell recordings, outward-rectifier potassium currents were larger in small-diameter DRG neurons from adult *Clcn3*^–/–^ mice than in those from adult WT mice (Figure 3E). To identify which types of K^+^ channels are affected by ClC-3 deletion, we recorded K^+^ currents before and during the application of 5 mM 4-AP. 4-AP blocks A-type fast-inactivating voltage-gated K^+^ currents (*I*_*A*_), whereas both slow-inactivating voltage-gated K^+^ currents (*I*_*D*_) and delayed-rectifier voltage-gated K^+^ currents (*I*_*K*_) are 4-AP-insensitive (Vydyanathan et al., 2005). *I*_*D*_ and *I*_*K*_ currents were larger in neurons from adult *Clcn3^–/–^* mice compared with adult WT mice (Figure 3F), whereas *I*_*A*_ currents did not differ (Figure 3G). DRG neurons also express inward-rectifier potassium channels (K_ir_2.3 and K_ir_2.4) (Busserolles et al., 2020) or K_ir_3.1 (Seitz et al., 2021). Electrophysiological recording of inward-rectifier potassium channels showed small currents of up to 300 pA at -160 mV (Supplementary Figure 4G), with no difference between WT and *Clcn3*^–/–^ DRG neurons (Supplementary Figure 4H). We conclude that ClC-3 regulates the plasma membrane density of slow-inactivating and delayed-rectifier voltage-gated K^+^ channels, but not of other K^+^ channels.

### ClC-3 Cl^–^/H^+^ transport is not required for the normal excitability of sensory dorsal root ganglion cells

ClC-3 exchanges Cl^–^ for H^+^ at a fixed stoichiometry of 2:1 and, thus, might modify endosomal [Cl^–^] or pH (Guzman et al., 2013; Rohrbough et al., 2018). To separate the function of ClC-3-associated Cl^–^/H^+^ exchange in regulating neuronal excitability from other ClC-3 functions, we used a knock-in animal model expressing mutant ClC-3 with a neutralizing point mutation in the proton glutamate: E281Q, E339Q, and E312Q in ClC-3a, ClC-3b, and ClC-3c, respectively (Accardi et al., 2005; Guzman et al., 2013; Rohrbough et al., 2018). In each isoform, this mutation abolishes Cl^–^/H^+^ exchange but does not affect the ability to heterodimerize with ClC-4 and to target ClC-4 to recycling endosomes or late endosome/lysosomes (Guzman et al., 2013, 2017). Since ClC-4 is also expressed in DRG neurons (Supplementary Figure 5), we generated the double-mutant *Clcn3*^E281Q/E281Q^*/Clcn4*^–/–^ to completely abolish ClC-3-associated Cl^–^/H^+^ exchange in all organelles. *Clcn4*^–/–^ and *Clcn3^E281Q/E281Q^* pups are born at the Mendelian ratio, are fertile, and develop normally with no obvious phenotype. In contrast, *Clcn3*^E281Q/E281Q^*/Clcn4*^–/–^ are born at the Mendelian ratio; they show a stronger phenotype than the *Clcn3^–/–^*, are smaller, and develop with delay in comparison to littermates.

**FIGURE 4:**
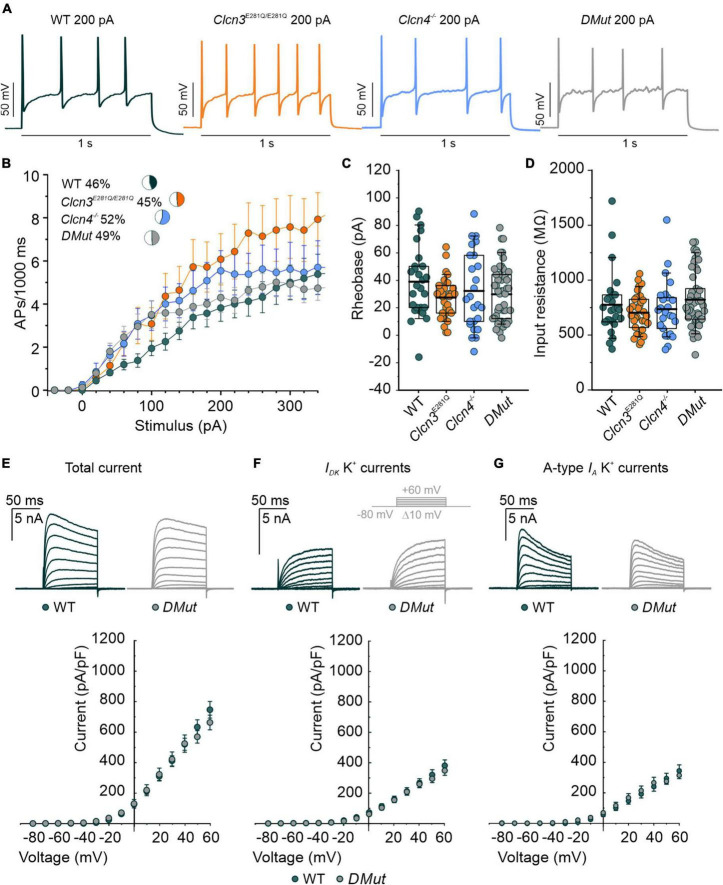
Comparison of the action potential (AP) properties of wild type (WT), *Clcn3^E281Q/E281Q^*, *Clcn4*^–/–^, and *Clcn3*^E281Q/E281Q^*/Clcn4*^–/–^ (*DMut*) dorsal root ganglion (DRG) neurons from adult mice. **(A)** Representative APs elicited by 200 pA current injection in adult WT (green), *Clcn3^E281Q/E281Q^* (orange), *Clcn4*^–/–^ (blue), and *DMut* (gray) neurons. **(B)** AP frequencies for all conditions. Insets show the percentage of multiple firing neurons for each condition. **(C)** Rheobase and **(D)** input resistance. Data were collected from WT (green, *n* = 27 cells from three animals), *Clcn3^E281Q/E281Q^* (orange, *n* = 31 cells from three animals), *Clcn4^–/–^* (blue, *n* = 25 cells from three animals), or *Clcn3*^E281Q/E281Q^*/Clcn4^–/––^* (gray, *n* = 49 from seven animals) neurons. **(E–G)** Representative recordings of the K^+^ current (upper panel) and current–voltage relationship (lower panel) were obtained from WT (green, *n* = 16 from five animals) and *DMut* (gray, *n* = 10 from three animals) DRG neurons. The inset illustrates the voltage protocol used to elicit K^+^ currents during and after 4-AP application. Total **(E)**, slow-inactivating **(F),** and fast-inactivating **(G)** K^+^ currents densities are not different between phenotypes. One-way ANOVA or one-way Kruskal–Wallis ANOVA was used for statistical analysis. Data are presented as the mean ± SEM. In boxplots, boxes indicate the upper and lower quartiles, and whiskers the upper and lower 90 percentiles.

For DRG neurons isolated from P60 animals, AP frequencies were similar for WT, *Clcn3*^E281Q/E281Q^*, Clcn4*^–/–^, and *Clcn3*^E281Q/E281Q^*/Clcn4*^–/–^ (Figures 4A,B). All AP parameters tested were indistinguishable between DRG neurons from P60 mutant and WT mice (Figures 4C,D and Supplementary Figure 6). Total potassium currents were similar between *Clcn3*^E281Q/E281Q^*/Clcn4*^–/–^ and WT cells. Furthermore, the separation of A-type fast-inactivating from slow-inactivating voltage-gated K^+^ channels confirmed the WT-like phenotype of *Clcn3*^E281Q/E281Q^*/Clcn4*^–/–^ neurons, with *I*_*A*_, *I*_*D*,_ and *I*_*K*_ currents similar to those of WT neurons (Figures 4E–G). These results suggest that ClC-3-associated Cl^–^/H^+^ exchange is not required for normal excitability in sensory DRG neurons.

### Cl^–^/H^+^ transport activity regulates microglia activation

**FIGURE 5:**
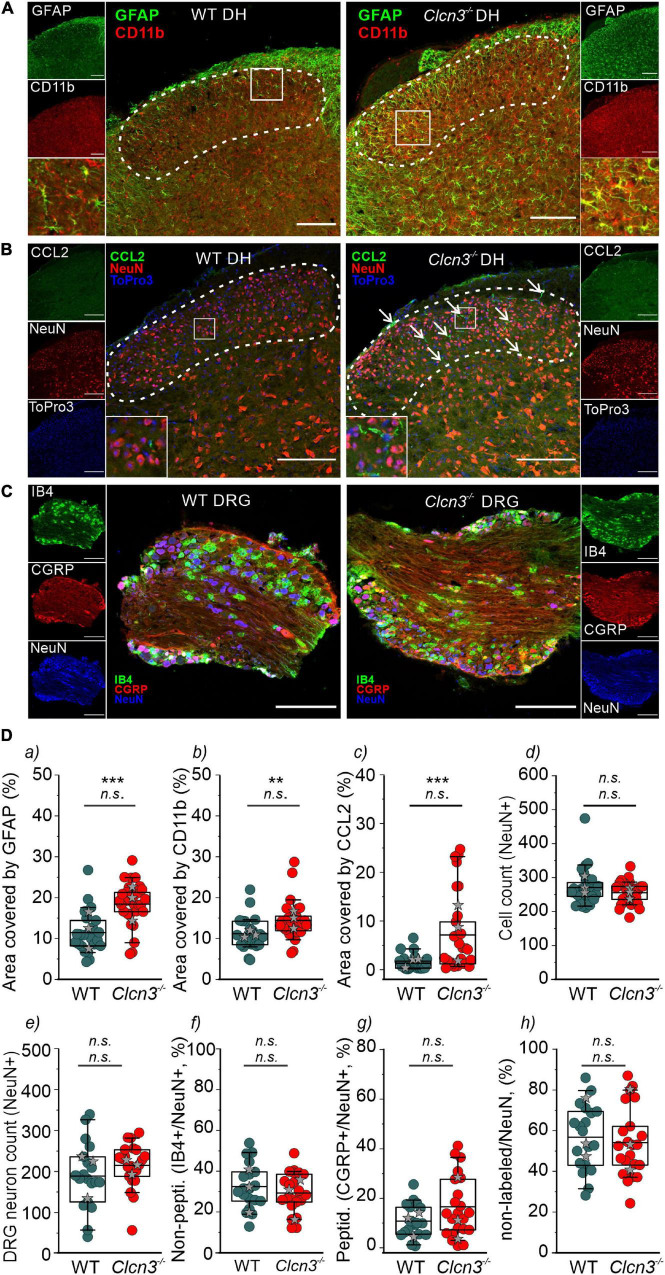
ClC-3 disruption leads to neuroinflammation within the DHSC. **(A)** glial fibrillary acidic protein (GFAP) and CD11b positivity in the dorsal horn (DH) layers I–IV of wild type (WT) and *Clcn3*^–/–^ mice. **(B)** Section of the lumbar spinal cord from WT and *Clcn3*^–/–^ mice stained for CCL2 (green), NeuN (red), and TO-PRO-3 (blue, nuclei). **(C)** Representative immunofluorescence confocal images of dorsal root ganglia from WT and *Clcn3*^–/–^ mice stained for NeuN (blue), IB4 non-peptidergic small-diameter (green), and CGRP peptidergic (red) neurons. **(D)** (a) Area of glia expressing GFAP, (b) activated microglia expressing CD11b, (c) area of neuronal CCL2 expression, and (d) neuronal count in the dorsal horn from WT and mutant spinal cords. (e–h) Quantitative analysis of the total neuron count within the dorsal root ganglion (DRG). Each dot represents a single measurement, (a–d) WT (*n* = 29), *Clcn3*^–/–^ (*n* = 33); (g, h) WT (*n* = 23), *Clcn3*^–/–^ (*n* = 29). Three or four mice were used per genotype, with at least six slices from the lumbar section of each animal. Sections were 20-μm thick and collected every 100 μm apart. Scale bar: 200 μm. Statistical significance levels are **p < 0.01, ***p < 0.001, n.s., not significant. *Clcn3*^–/–^ differs from WT using the Student’s *t-*test (upper symbol), and statistics per animal with the data displayed with gray stars (lower symbol). In boxplots, boxes indicate the upper and lower quartiles, and whiskers the upper and lower 90 percentiles.

Microglia are a specialized population of macrophage-like cells in the CNS that modulate neuronal activity and neuronal excitability in nociceptive pathologies (Vallejo et al., 2010). Immunohistochemical analysis with antibodies against the microglial marker integrin alpha-M or also known as cluster of differentiation molecule 11B (CD11b) and the astrocyte marker glial fibrillary acidic protein (GFAP) (Lan et al., 2017) was conducted to evaluate glial activation in *Clcn3^–/–^* from adult mice (Figure 5A, dashed line, and Figure 5Da,b and Supplementary Figure 7). In layers I, II, III, and IV of the DHSC of P60 animals, GFAP levels were increased by about 89% and CD11b levels by 49% in *Clcn3*^–/–^ compared with WT (Figure 5Da,b). In P21 *Clcn3*^–/–^ mice, microglia and astrocyte proliferation were increased (Supplementary Figure 8). These results indicate that neuroinflammatory changes are associated with glial activation in the dorsal horn of the *Clcn3*^–/–^ spinal cord.

Chemokine ligand 2 is a chemotactic factor that attracts immune cells, such as monocytes/macrophages and natural killer cells. Increased CCL2 expression in DRG neurons and the spinal cord has been associated with inflammatory pain and hypersensitivity (Menetski et al., 2007; Illias et al., 2018; Gschwandtner et al., 2019). At P60, fine cellular processes reactive to CCL2 were readily visible and more abundant within the DHSC region in *Clcn3*^–/–^ than in WT mice (Figure 5B), resulting in larger CCL2-stained areas in the *Clcn3*^–/–^ dorsal horn (Figure 5Dc). These observations are consistent with increased CCL2 secretion contributing to the development of hyperalgesia in *Clcn3*^–/–^. To test for neurodegeneration, dorsal root ganglia or spinal cord sections were stained for the neuronal marker NeuN. This revealed no difference in the total number of neurons between the dorsal root ganglia of P60 WT and mutant animals (Figures 5C,De). Isolectin B4 (IB4) is a marker for non-peptidergic neurons, and calcitonin gene-related peptide (CGRP) is a marker for peptidergic neurons (Priestley, 2009). Staining for IB4 and CGRP revealed more non-peptidergic IB4-positive cells than peptidergic CGRP-positive cells (Figure 5Df,g) in both WT and *Clcn3^–/–^* mice, but no differences in the number of cells between WT and mutant tissues. Results were similar for large- and medium-diameter neurons, which are NeuN-positive, but negative for IB4 and CGRP (Figure 5Dh). The DHSC receives nociceptive inputs in layers I and II and non-nociceptive inputs in layers I, IV, and V from small-, medium-, and large-diameter DRG neurons (Stucky, 2007). Similar to the dorsal root ganglia, neuron numbers in layers I–IV of the DHSC were not reduced in *Clcn3^–/–^* compared with WT animals (Figure 5Dd). We conclude that genetic ablation of ClC-3 does not lead to neurodegeneration in the dorsal root ganglia or in the dorsal horn of the spinal cord.

**FIGURE 6:**
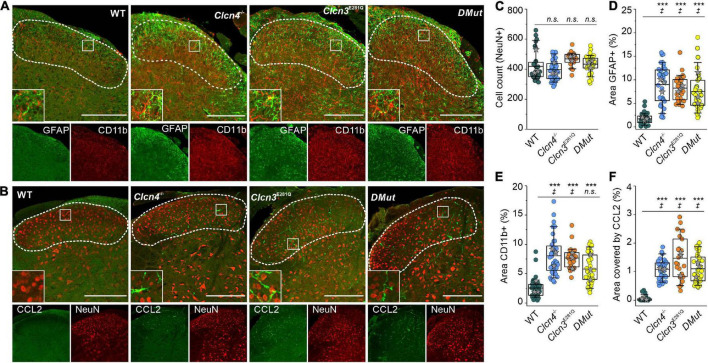
Reduced Cl^–^/H^+^ exchange triggers neuroglia activation in the spinal cord. **(A)** Representative immunofluorescence confocal images of lumbar spinal cord sections from WT, *Clcn3^E281Q/E281Q^*, *Clcn4*^–/–^, and *Clcn3*^E281Q/E281Q^*/Clcn4*^–/–^ (*DMut*) mice stained for glial fibrillary acidic protein (GFAP) (green) and CD11b (red). Layers I–IV of the dorsal horn are outlined with a dashed line. Insets show increased GFAP and CD11b reactivity in mutant mice. **(B)** Lumbar spinal cord sections from mice with the indicated genotypes were stained for CCL2 (green) and NeuN (red). **(C)** Total cell numbers in the dorsal horn of WT and mutant’s spinal cords. **(D)** Areas of glia positive for GFAP or to CD11b in **E** for WT (*n* = 34), *Clcn3^E281Q/E281Q^* (*n = 24*), *Clcn4*^–/–^ (*n* = 36), and *Clcn3*^E281Q/E281Q^*/Clcn4*^–/–^ (*DMut*; *n* = 30). **(F)** Area of CCL2 immunofluorescence in WT (*n* = 30), *Clcn3^E281Q/E281Q^* (*n* = 23), *Clcn4*^–/–^ (*n* = 34), and *Clcn3*^E281Q/E281Q^*/Clcn4*^–/–^ (*n* = 36). Each dot represents a single measurement. Three or four mice were used per genotype, with at least six slices from the lumbar section of each animal. Sections were 20-μm thick and collected every 100 μm apart. Scale bar: 200 μm. Asterisk (*) was used to represent differences between groups and (^‡^) when also animals were compared (^‡^*p* < 0.05, ***p < 0.001). n.s., not significant; one-way ANOVA (Tukey’s HSD *post hoc* test). In boxplots, boxes indicate the upper and lower quartiles, and whiskers the upper and lower 90 percentiles.

To investigate the role of ClC-3-associated Cl^–^/H^+^ exchange in these inflammatory processes, we compared GFAP and CD11b levels in WT, *Clcn3^E281Q/E281Q^*, *Clcn4*^–/–^, and *Clcn3*^E281Q/E281Q^*/Clcn4*^–/–^ mice (Figure 6). Numbers of neuronal cells were similar (Figure 6C), and areas reactive to GFAP or positive for the microglia marker CD11b were significantly increased in all genotypes (Figures 6A,D,E). Moreover, glial cells had more and longer processes in mutants than in WT mice (Figures 6A,B, insets). The CCL2-positive area was ∼30-fold greater in *Clcn3^E281Q/E281Q^*, *Clcn4*^–/–^, and *Clcn3*^E281Q/E281Q^*/Clcn4*^–/–^ than in WT mice (Figures 6B,F). Overall, these findings demonstrate that fully functional ClC-3 and ClC-4 chloride transporters are required to prevent microglia and astrocyte proliferation.

### Neurodegeneration in *Clcn3*^–/–^, *Clcn3^E281Q/E281Q^*, *Clcn4*^–/–^, and *Clcn3^E281Q/E281Q^*/*Clcn4*^–/–^ animals

**FIGURE 7:**
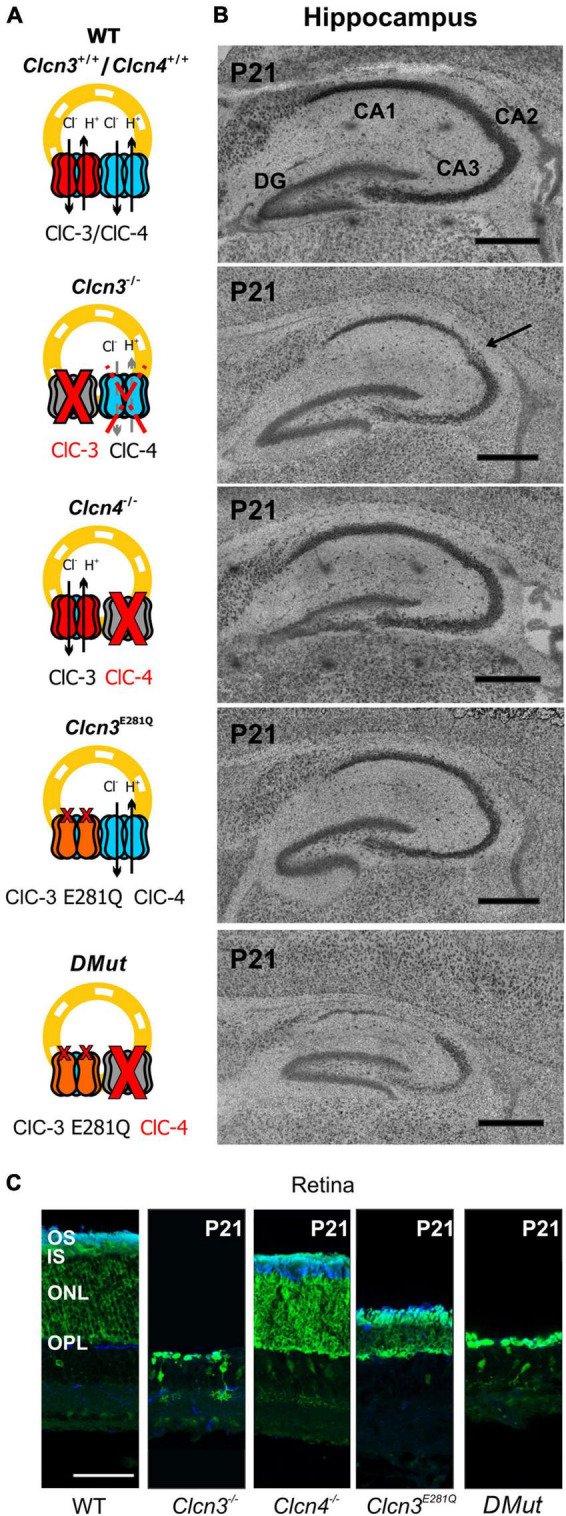
Hippocampal and retinal degeneration in different animal models of Cl^–^/H^+^ exchange. **(A)** Cartoons illustrating heterodimerization between ClC-3 and ClC-4 and the contribution of Cl^–^/H^+^ transport function in endosomes for each genotype and **(B)** representative confocal images of Nissl-stained sagittal (100 μm) section indicating hippocampal formation from 21-day-old WT, *Clcn3*^–/–^, *Clcn3^E281Q/E281Q^*, *Clcn4*^–/–^, and *Clcn3*^E281Q/E281Q^*/Clcn4*^–/–^ (*DMut*) mice. The arrow indicates neurodegeneration in the CA2 region of the *Clcn3*^–/–^ hippocampus. **(C)** Representative immunofluorescence confocal images of retinal sections from different mouse genotypes at P21. Sections were immunostained using an anti-recoverin antibody to indicate the inner segments, somas, and terminals of photoreceptors (green; this antibody also occasionally labels type 2 bipolar cells) and anti-rhodopsin to identify the outer segment of rod cells (blue). OS, outer segment; IS, inner segment; ONL, outer nuclear layer; OPL, outer plexiform layer. Scale bar: 0.5 mm for hippocampal and 50 μm for retinal images.

Hippocampal and retinal neurodegeneration is well established in *Clcn3^–/–^* animals (Stobrawa et al., 2001; Dickerson et al., 2002). The lack of neurodegeneration in the DRG or DHSC region (Figure 5) indicates that separate classes of neurons have distinct sensitivities to reduced levels of Cl^–^/H^+^ exchange. This finding prompted us to study neurodegeneration in the hippocampus and retina in various animal models with distinct levels of endosomal Cl^–^/H^+^ exchange (Figure 7). In agreement with previous studies, ClC-3 ablation resulted in severe neurodegeneration in the hippocampus and retina (Figure 7; Stobrawa et al., 2001; Dickerson et al., 2002). *Clcn4^–/–^* mice do not exhibit hippocampal or retinal degeneration (Figure 7). *Clcn3^E281Q/E281Q^* animals had normal hippocampal morphology but the reduced thickness of the photoreceptor layer (Figure 7C), indicating that the retina is more sensitive than the hippocampus to reduced levels of ClC-3-associated Cl^–^/H^+^ exchange. CA1 and CA2 hippocampal regions and retinal tissue in *Clcn3*^E281Q/E281Q^*/Clcn4*^–/–^ mice were almost completely absent at the age of P21 (Figures 7A,B). These findings highlight the sensitivity of neuronal tissues to reduced Cl^–^/H^+^ exchange. Whereas the DHSC is viable in the complete absence of ClC-3-associated transport in *Clcn3^–/–^*, the retina is degenerated by the absence of ClC-3-mediated Cl^–^/H^+^-exchange even in presence of ClC-4. The hippocampus tolerates reduced Cl^–^/H^+^-exchange in *Clcn3^E281Q/E281Q^*, but is complete loss in *Clcn3*^E281Q/E281Q^*/Clcn4*^–/–^ and *Clcn3*^–/–^.

## Discussion

We have demonstrated that genetic ablation of the Cl^–^/H^+^ exchanger ClC-3 alters nociception in mice. In *Clcn3*^–/–^ animals, we observed enhanced electrical activity of peripheral nociceptors and inflammatory processes at the spinal cord. We considered that ClC-3 might affect cellular processes either by changing chemical or electrical gradients at endosomes or by supporting the intracellular trafficking of other proteins *via* a chaperone-like function. To distinguish between these possibilities, we generated mutant mice (*Clcn3^E281Q/E281Q^*) that express transport-incompetent ClC-3. Since E281Q ClC-3 can still heterodimerize with the related transporter ClC-4, the genetic ablation of ClC-4 was also necessary to abolish all ClC-3-associated Cl^–^/H^+^ exchanges in recycling endosomes and lysosomes. *Clcn3*^E281Q/E281Q^*/Clcn4*^–/–^ animals had an inflammatory response in the DHSC and early onset hippocampal and retinal degeneration, illustrating the importance of ClC-3 Cl^–^/H^+^ transport for neuroglia function and neuronal integrity in supraspinal brain regions. In contrast, the electrical properties of sensory DRG neurons were similar in *Clcn3^E281Q/E281Q^* and *Clcn3*^E281Q/E281Q^*/Clcn4*^–/–^ mice, indicating that ClC-3-dependent regulation of neuronal excitability is independent of Cl^–^/H^+^ exchange.

ClC-3 ablation impairs age-related adjustment in neuronal excitability, which is thought to result from developmental changes in the expression and/or function of ion channels, such as Na^+^ and K^+^ channels (Scott et al., 1988; Wang and Albers, 2009). Specifically, reduced K^+^ conductances were proposed to reduce excitability in old neurons (Scott et al., 1988). DRG neurons from adult *Clcn3*^–/–^ (but not *Clcn3*^E281Q/E281Q^*/Clcn4*^–/–^ mice) had increased densities of slow-inactivating and delayed-rectifier voltage-gated K^+^ currents (Figure 3F). Other current components, such as *I*_*A*_ and inward-rectifying voltage-gated K^+^ currents, were unaffected by ClC-3 ablation. Two-pore domain K^+^ (K_2P_) channels help to set and stabilize the RMP (Rasband et al., 2001). Since the RMP was unchanged, we excluded the possibility that the expression or trafficking of K_2P_ channels is altered in *Clcn3*^–/–^ mice. A greater number of voltage-dependent K^+^ channels in knockout cells may facilitate AP repolarization and enhance excitability (Scott et al., 1988). We found a reduced density of TTX-R Na^+^ currents mediated by Na_*v*_1.8 and/or Na_*v*_1.9 in DRG neurons from adult *Clcn3*^–/–^ mice (Figure 3C), but no significant change in TTX-S currents (Figure 3D), which are predominately mediated by Na_*v*_1.7 (Chen et al., 2018). Modeling studies have demonstrated that the excitability of peripheral neurons depends on the ratio of Na_*v*_1.7 to Na_*v*_1.8 (Petersson et al., 2014; Tigerholm et al., 2015); thus, the observed changes in sodium currents can explain the observed hyperexcitability of *Clcn3^–/–^* DRG. TTX-R currents play a dominant role in all phases of the AP (Blair and Bean, 2002) and are believed to adjust the AP voltage threshold in DRG neurons (Bennett et al., 2019). Our results indicate that ClC-3 contributes to the homeostatic regulation of neuronal excitability by controlling the number of ion channels, such as Na^+^ and K^+^ channels in the surface membrane. Since these changes were not found in *Clcn3*^E281Q/E281Q^*/Clcn4*^–/–^ mice, we conclude that the regulatory mechanism is not mediated by Cl^–^/H^+^ exchange activity but instead by a chaperone function of ClC-3.

Formalin injection induces a biphasic pain reaction (Figure 1). During the early phase, activation of C-type nociceptors and the release of mediators, such as ATP, glutamate, kinins, histamine, serotonin, cytokines, and tropic factors, promote peripheral sensitization (Hunskaar and Hole, 1987; Amaya et al., 2013). The second phase is dominated by inflammatory responses in the DHSC (Hunskaar and Hole, 1987; Tjølsen et al., 1992). Young and adult *Clcn3*^–/–^ mice were more sensitive to formalin application. This enhanced behavioral pain response during the second phase suggests that a central sensitization within the dorsal horn changes the elicited sensory response and releases pro-inflammatory mediators to the spinal cord *in Clcn3*^–/–^ (Ren and Dubner, 2008; Vallejo et al., 2010; Mika et al., 2013). We found increased numbers of activated astrocytes and microglia within the spinal cord of *Clcn3*^–/–^ mice at P21 and P60 (Supplementary Figure 8; Figure 5). The identification of reactive microglia in young and adult *Clcn3*^–/–^ and in adult *Clcn4*^–/–^, *Clcn3^E281Q/E281Q^*, and *Clcn3*^E281Q/E281Q^*/Clcn4*^–/–^ mice (Figure 5; and Figure 6) suggests that astrocytes and microglia homeostasis critically depend on the presence of fully competent, functional chloride transporters.

Chemokine ligand 2 is a chemotactic factor that attracts monocytes, CD4+ T cells, natural killer cells, and dendritic cells to sites of inflammation (Carr et al., 1994). Increased CCL2 expression following inflammation or injury attracts leukocytes to mediate defense, cytokine release, and repair (Gschwandtner et al., 2019). Higher CCL2 expression in DRG neurons, as well as in the spinal cord, is associated with inflammatory pain and hypersensitivity (Menetski et al., 2007; Illias et al., 2018). We studied the effect of ClC-3-associated Cl^–^/H^+^ exchange on CCL2 expression levels in dorsal horn layers I–IV and found that CCL2 levels were increased in *Clcn3*^–/–^, *Clcn4*^–/–^, *Clcn3^E281Q/E281Q^*, and *Clcn3*^E281Q/E281Q^*/Clcn4*^–/–^ mice (Figures 5B,Dc, 6B,F). A recent report demonstrated that CC12 is released *via* recycling endosomes and that changes in endo-lysosomal ion transport directly stimulate CCL2 release by promoting transfer through early and recycling endosomes (Plesch et al., 2018). ClC-3 isoforms are found in different organelles of the endo-lysosomal trafficking system, with ClC-3c present in recycling endosomes (Guzman et al., 2015; Comini et al., 2022). Taken together, our data suggest that impaired endosomal Cl^–^/H^+^ exchange causes neuroinflammation and pain in the *Clcn3*^–/–^ mouse model *via* enhanced CCL2 release triggering an inflammatory reaction.

Many ion channels are sorted *via* the secretory pathway (Griffith, 2001; Swanwick et al., 2010; Capera et al., 2019) and recycled *via* clathrin-mediated endocytosis (Okuse et al., 2002; Liu et al., 2005; Swanwick et al., 2010; Conrad et al., 2018, 2021). ClC-3 has clathrin-binding motifs in its amino terminus (Zhao et al., 2007; Stauber and Jentsch, 2010) and, thus, might facilitate the endocytosis of membrane proteins and direct them to lysosomes (ClC-3b) or the recycling endosome (ClC-3c) (Guzman et al., 2015, 2017; Comini et al., 2022). In *Clcn3*^E281Q/E281Q^*/Clcn4*^–/–^ mice, ClC-3-associated endosomal Cl^–^/H^+^ exchange does not occur. In contrast, *Clcn3^E281Q/E281Q^* animals have no obvious phenotype, likely because E281Q ClC-3 can still interact with and direct ClC-4 to the recycling endosome and lysosome (Guzman et al., 2017; Weinert et al., 2020). There are no phenotypic alterations in *Clcn4*^–/–^, but the lack of ClC-4 in the *Clcn3*^E281Q/E281Q^*/Clcn4*^–/–^ mouse caused a strong CNS phenotype (Figure 7), with degenerated hippocampal CA1 and CA2 regions at the age, at which neurodegeneration is starting in the *Clcn3*^–/–^ mouse. Retinal tissue appears to be more sensitive to reduced levels of Cl^–^/H^+^ transporters than the hippocampus. Whereas hippocampal regions are not affected in the *Clcn3^E281Q/E281Q^*, the reduced thickness of retinal neuronal tissue in these animals already evidences the tight dependency between the viability of the neurons and Cl^–^/H^+^ transporters in this CNS region. Recently, the *Clcn3*^unc/unc^ knock-in mouse model was generated, carrying the E224A mutation in the “gating glutamate” that uncouples Cl^–^ currents from H^+^ counter transport. *Clcn3*^unc/unc^ mice do not have a severe phenotype, but in *Clcn3*^unc/unc^/*Clcn4*^–/–^ animals hippocampal neurodegeneration can be seen in the third postnatal week (Weinert et al., 2020). Data from the *Clcn3*^E281Q/E281Q^*/Clcn4*^–/–^ and *Clcn3*^unc/unc^/*Clcn4*^–/–^ mouse models reinforce the importance of ClC-3 Cl^–^/H^+^ transport function for neuronal survival within the CNS in supraspinal brain regions, such as the hippocampus and retina.

**FIGURE 8:**
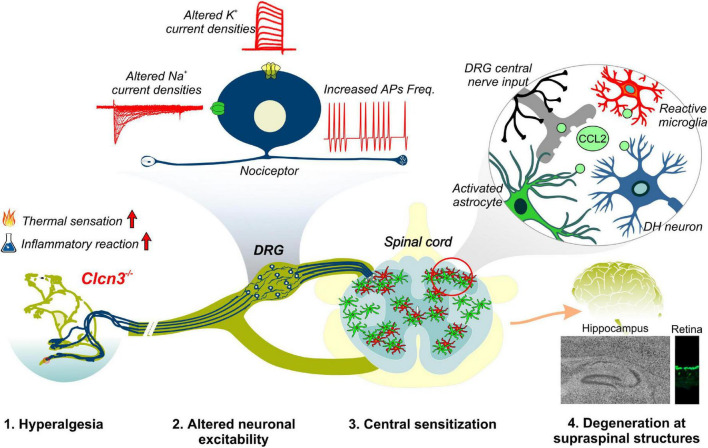
Summary of phenotypic alterations in *Clcn3*^–/–^.

Ablation or mutation of the genes encoding CLC-type Cl^–^/H^+^ exchangers has been linked to a variety of neurodevelopmental syndromes. Patients with these syndromes often have severe symptoms, thought to be caused by disturbed endosomal ion homeostasis. Here, we identified ClC-3-dependent cellular processes that are not coupled to its ion-exchange mechanism but are instead linked to a possible chaperone function. Our findings, summarized in Figure 8, identified ClC-3 as an integral part of the molecular machinery underlying age-related changes in neuronal sensitivity and excitability. We also demonstrated that altered Cl^–^/H^+^ transport may modify chemokine release and, thus, regulate inflammatory processes. Modulation of inflammatory responses by CLC-type transporter functions may be responsible for the severe phenotype of engineered animal models and may contribute to symptom severity in patients with CLC-linked neurological diseases.

## Data availability statement

The original contributions presented in this study are included in the article/Supplementary material, further inquiries can be directed to the corresponding author.

## Ethics statement

The animal study was reviewed and approved by German Animal Welfare Act (TierSchG § § 7–9) and State Agency for Nature, Environment and Consumer Protection, North Rhine Westphalia, and the local Animal Protection Committee, file numbers 84-02.04.2015.A108 and 84-02.04.2015.A307.

## Author contributions

JS-M performed the experiments, analyzed the data, and drafted the manuscript. AW, CB, and MS performed the animal behavior test and analyzed the data. SB-P performed the Western blot experiments. JG and FM performed the retina and hippocampal immunostainings experiments. AL provided the macro for VGSC analyses. CF supervised the research and wrote the manuscript. RG conceived the idea, supervised the research, and wrote the manuscript. All authors contributed to the article and approved the submitted version.
