# Corrigendum: ClC-3 regulates the excitability of nociceptive neurons and is involved in inflammatory processes within the spinal sensory pathway

**DOI:** 10.3389/fncel.2023.1186435

**Published:** 2023-04-13

**Authors:** Juan Sierra-Marquez, Antje Willuweit, Michael Schöneck, Stefanie Bungert-Plümke, Jana Gehlen, Carina Balduin, Frank Müller, Angelika Lampert, Christoph Fahlke, Raul E. Guzman

**Affiliations:** ^1^Institute of Biological Information Processing, Molecular and Cellular Physiology (IBI-1), Forschungszentrum Jülich, Jülich, Germany; ^2^Medical Imaging Physics, Institute of Neuroscience and Medicine (INM-4), Forschungszentrum Jülich, Jülich, Germany; ^3^Institute of Physiology, RWTH Aachen, Aachen, Germany

**Keywords:** ClC-3, chloride-proton exchanger, neuronal excitability, pain, microglia activation, action potential, DRG

In the original article, there was an error in the author list, and authors Antje Willuweit and Jana Gehlen were erroneously ordered. The corrected author list appears below.

Juan Sierra-Marquez, Antje Willuweit, Michael Schöneck, Stefanie Bungert-Plümke, Jana Gehlen, Carina Balduin, Frank Müller, Angelika Lampert, Christoph Fahlke and Raul E. Guzman

The authors apologize for this error and state that this does not change the scientific conclusions of the article in any way. The original article has been updated.

