# Supplementary material for: ClC-3 regulates the excitability of nociceptive neurons and is involved in inflammatory processes within the spinal sensory pathway
Source: Front Cell Neurosci. 2022 Aug 24;16:920075. doi: 10.3389/fncel.2022.920075 (PMC10134905; doi:10.3389/fncel.2022.920075)
Supplement: Supplementary file 1 [file Data_Sheet_1.pdf]

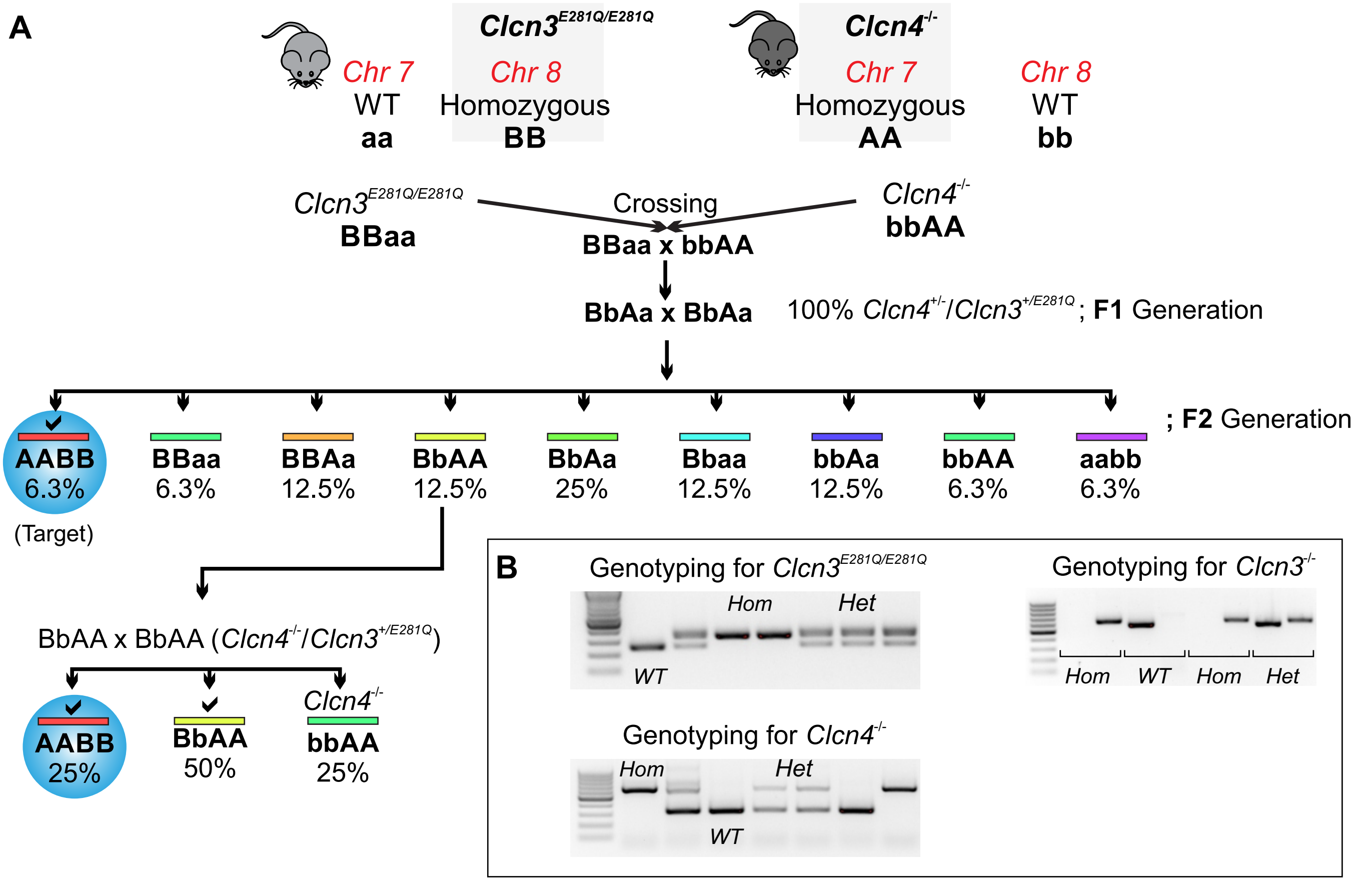


**Supplementary Fig 1.** Breeding strategy to generate *Clcn4*$^{-/-}$/*Clcn3*$^{E281Q/E281Q}$ double mutant mice. **A)** six males and females from each condition were crossed to generate five different families of the first generation (F1) *Clcn4*$^{-/+}$/*Clcn3*$^{E281/+}$ mice. Crossing F1 animals generated nine different genotypes. The double mutant line was maintained as male or female *Clcn4*$^{-/-}$/*Clcn3*$^{E281/+}$. **B)** Agarose gels of the different genotype reactions for *Clcn4*$^{-/-}$ (WT band 329bp and mutant 628bp), *Clcn3*$^{E281Q/E281Q}$ (WT band 250bp and mutant 332bp), and *Clcn3*$^{-/-}$ (WT band 550bp and mutant 650bp, performed separately).

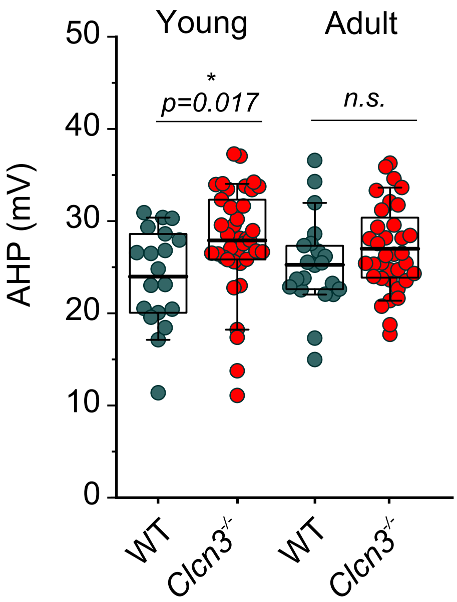


**Supplementary Fig 2.** After-hyperpolarization amplitude (AHP) of the action potential in WT and mutant DRG neurons. AHP in young and adult DRG neurons from WT (green) and *Clcn3*$^{-/-}$ (red) cells: young, WT (*n*=19 cells from four animals) and *Clcn3*$^{-/-}$ (*n*=38 cells from four animals); adult, WT (*n*=21 cells from seven animals) and *Clcn3*$^{-/-}$ (*n*=36 cells from six animals). Statistical significance levels *$p$<0.05, n.s., not significant; one-way ANOVA (Tukey's HSD post hoc test). In boxplots, boxes indicate the upper and lower quartiles, and whiskers the upper and lower 90 percentiles.

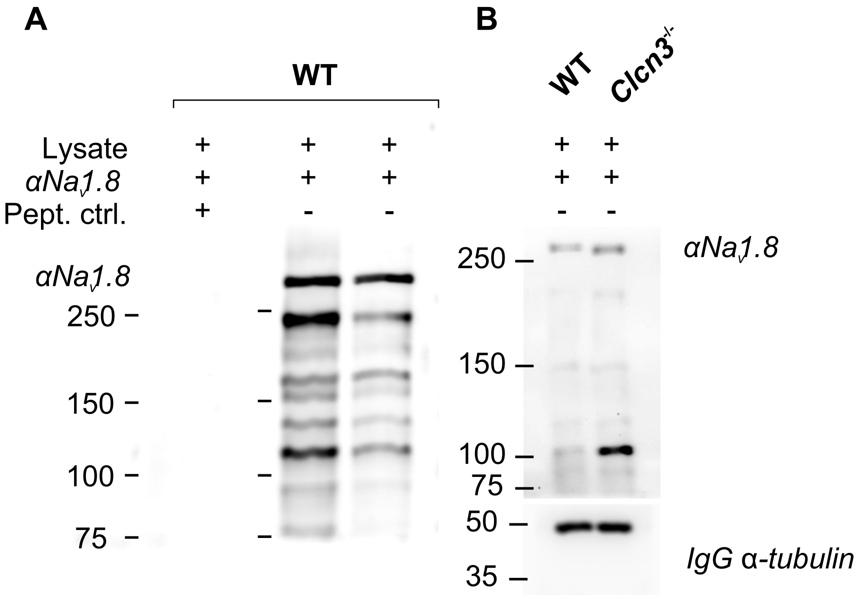


**Supplementary Fig 3.** Sodium channel $Na_v1.8$ protein expression in DRG neurons from WT and *Clcn3*$^{-/-}$ mice. (A) Representative western blots of a control experiment with lanes 1 and 2 loaded with 30 µg, and lane 3 with 15 µg of total membrane protein into a 7.5% acrylamide gel. After transfer to the PVDF membrane, only the first lane was incubated with a mixture of a 1:1 $Na_v1.8$ antibody and blocking peptide control, which is the amino acid sequence recognized by the antibody. The absence of a signal indicates that the antibody is bound to the peptide preventing targeting of the antibody to the sample. (B) Comparison of $Na_v1.8$ expression levels in lysates from dorsal roots ganglia of WT (*n*=3) and *Clcn3*$^{-/-}$ (*n*=3) mice. In this experiment, a total of 7 µg protein was loaded for each sample. The band at around 260 kDa corresponds to the whole size of the $Na_v1.8$ sodium channel.

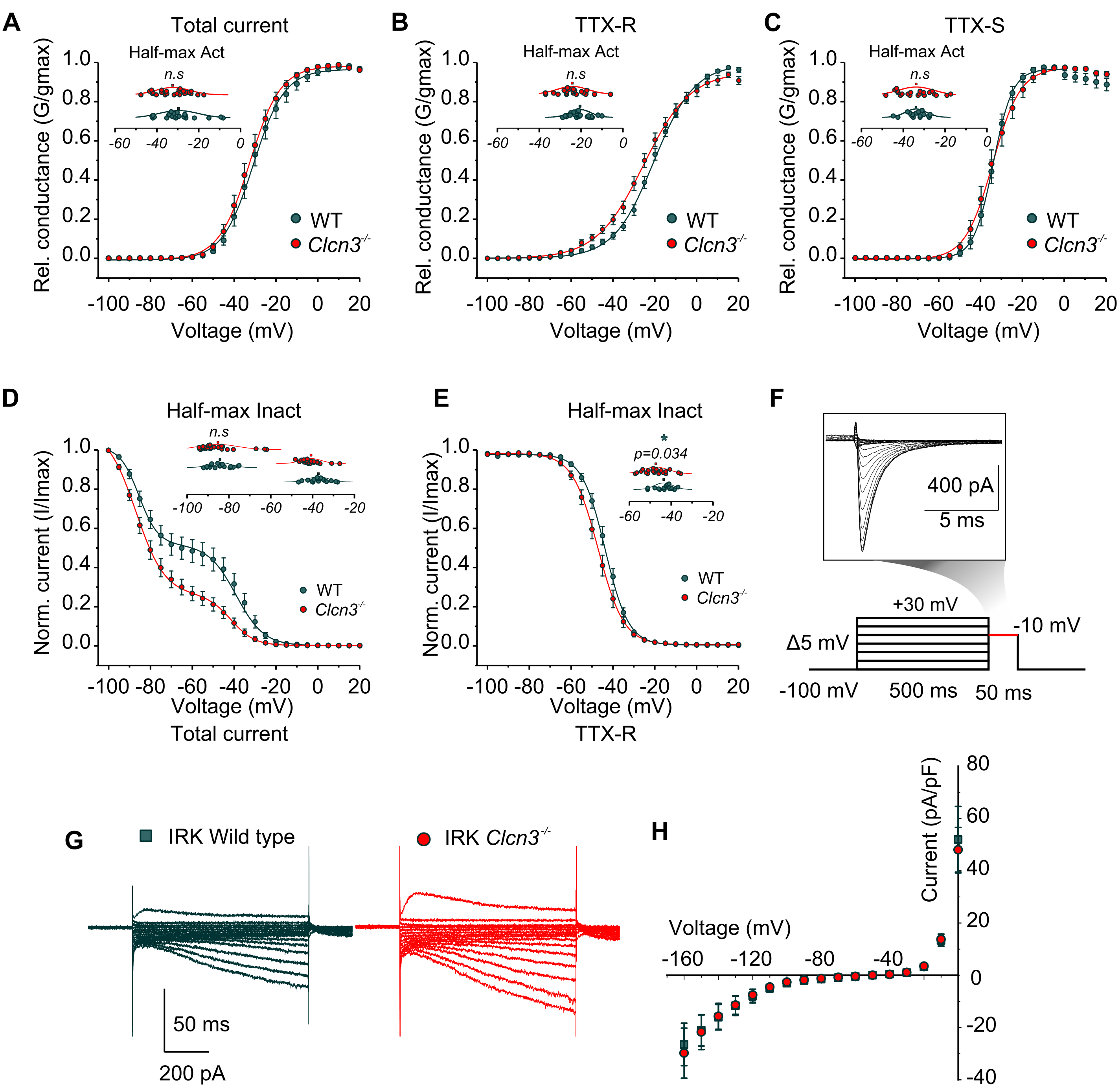


**Supplementary Fig 4.** Biophysical properties of $Na_v$ channels and inward-rectifying $K^+$ channels from small-diameter DRG adult neurons. (**A–C**) Voltage dependence of activation in WT and *Clcn3*$^{-/-}$ cells, fitted with single Boltzmann equations for total $Na^+$ currents (**A**), TTX-R currents (**B**), and TTX-S currents (**C**). Insets display the half-maximum activation for each cell. (**D–E**) Steady-state inactivation curves for total $Na^+$ currents fitted with a double Boltzmann equation (**D**) or for the TTX-R current component fitted with a single Boltzmann equation (**E**). Insets display the half-maximum voltage of inactivation for each cell. (**F**) Representative trace and voltage-clamp protocol to measure sodium current inactivation, consisting of a 500-ms test pulse between -100 mV and +30 mV, followed by a short (50 ms) pulse at -10 mV: WT (green; $n$=18 cells from five animals) and *Clcn3*$^{-/-}$ (red; $n$=22 cells from eight animals). (**G**) Representative recordings of inward-rectifying potassium (IRK) currents and (**H**) current–voltage-relationships for WT (green; $n$=15 cells from five animals) and *Clcn3*$^{-/-}$ (red; $n$=24 cells from eight animals) DRG neurons. Statistical significance level *$p$<0.05; Student's $t$-test. Data are presented as mean ± SEM. Half-max, half-maximum; Norm., normalized; Rel., relative.

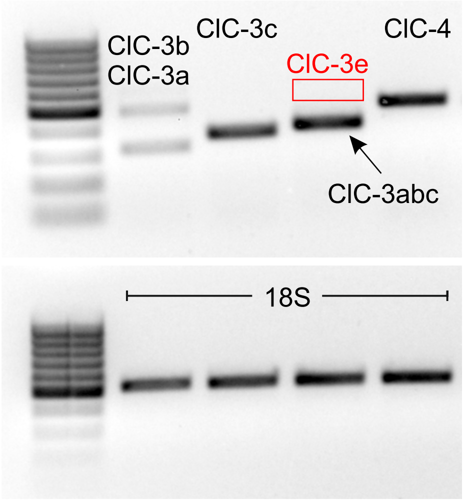


**Supplementary Fig 5.** Expression of different ClC-3 splice variants and ClC-4 in adult DRG neurons. Real-time PCR confirms the presence of three ClC-3 splice variants 3a, 3b, and 3c; ClC-3e (red) was not detected in the DRG of adult mice. 18S RNA was the control. Samples were collected from three adult mice.

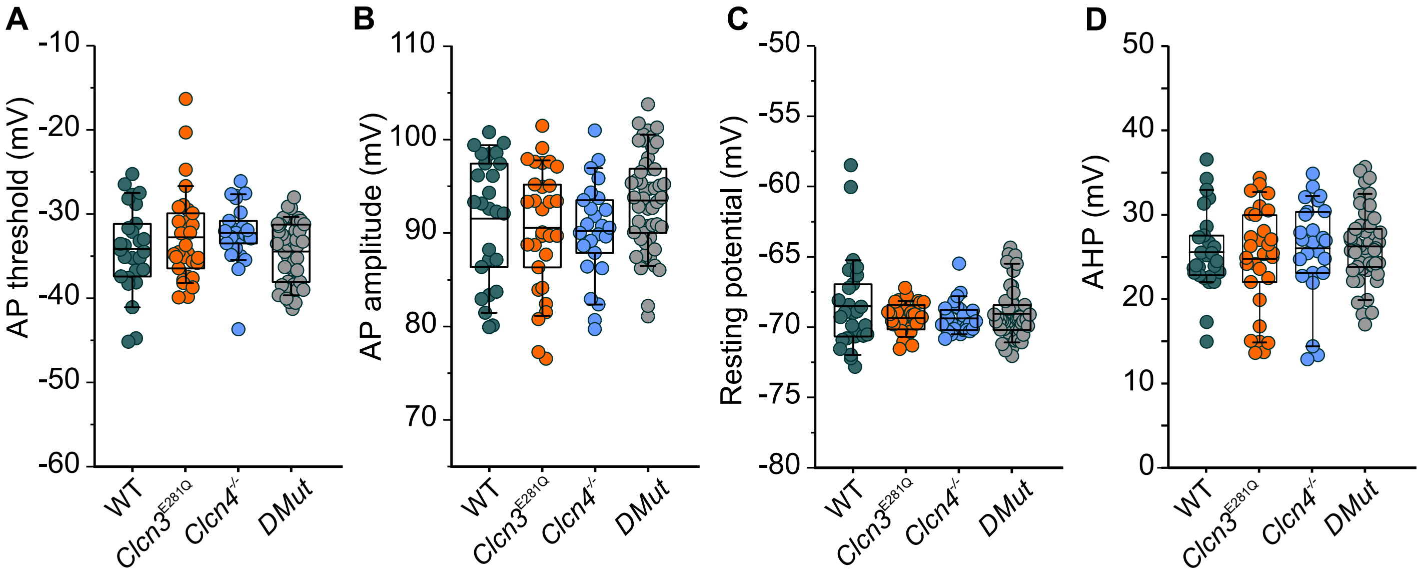


**Supplementary Fig 6.** Properties of action potential in DRG neurons with defective $Cl^-/H^+$ transport activity. (**A**) AP threshold, (**B**) AP amplitude, (**C**) resting membrane potential, and (**D**) AHP amplitudes from WT (green; *n*=27 cells from three animals), *Clcn3*$^{E281Q/E281Q}$ (orange, *n*=31 cells from three animals), *Clcn4*$^{-/-}$ (blue, *n*=25 cells from three animals), and *Clcn3*$^{E281Q/E281Q}$/*Clcn4*$^{-/-}$(*DMut*; gray, *n*=49 *cells* from seven animals) DRGs of adult mice. No differences were found between groups using one-way ANOVA. In boxplots, boxes indicate the upper and lower quartiles, and whiskers the upper and lower 90 percentiles.

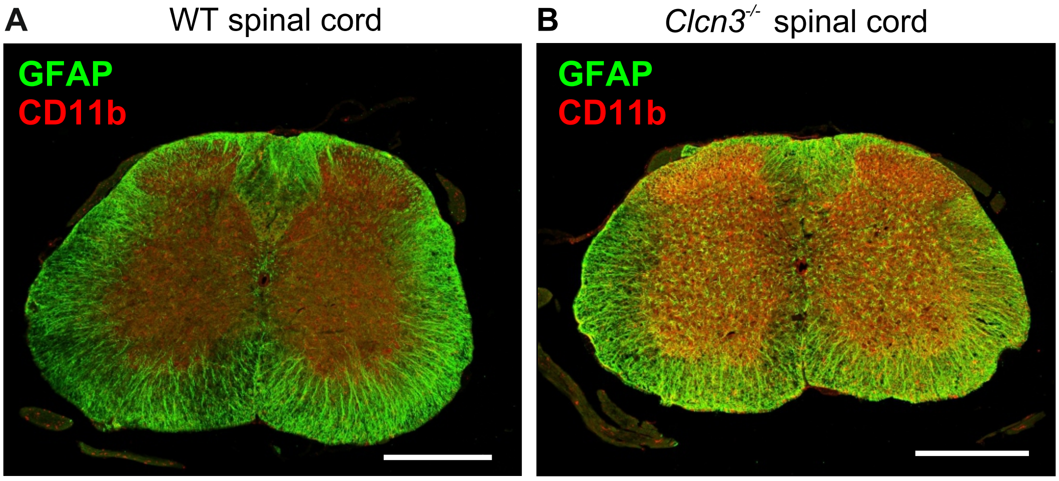


**Supplementary Fig 7.** CD11b and GFAP expression in P60 *Clcn3*$^{-/-}$ animals. (**A, B**) Representative confocal pictures of spinal cord sections showing astrocytes and microglia proliferation in adult mice from WT (**A**) and *Clcn3*$^{-/-}$ (**B**) animals. Sections were stained for GFAP (green) and CD11b (red). Scale bar: 200 μm. Slices were obtained from the lumbar section of the spinal cord using tile-scan scanning.

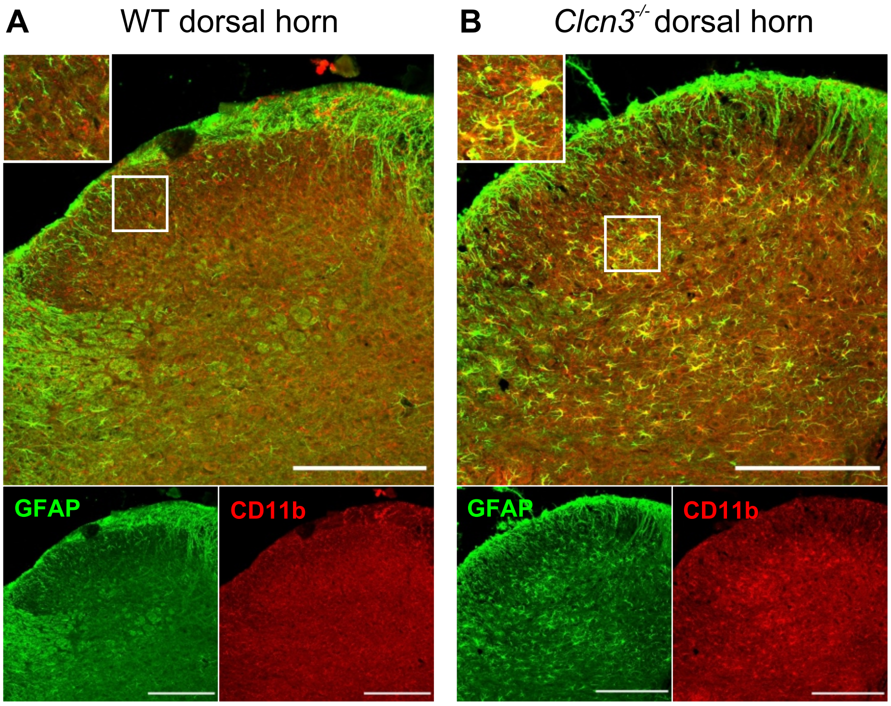


**Supplementary Fig 8.** Neuroglia homeostasis in young (P21) *Clcn3*$^{-/-}$ mice. (**A, B**) Representative confocal images of the DHSC, showing astrocytes and microglia proliferation in young WT (**A**) and *Clcn3*$^{-/-}$ (**B**) animals after staining for GFAP (green) and CD11b (red). Insets show morphological changes in the glia from both channels in *Clcn3*$^{-/-}$ as compared to the WT. Slices were obtained from the lumbar section of the spinal cord. Scale bar: 200 μm.
