# Supplementary material for: ClC-3 regulates the excitability of nociceptive neurons and is involved in inflammatory processes within the spinal sensory pathway
Source: Front Cell Neurosci. 2022 Aug 24;16:920075. doi: 10.3389/fncel.2022.920075 (PMC10134905; doi:10.3389/fncel.2022.920075)
Supplement: Supplementary file 2 [file Data_Sheet_2.pdf]

Condition	Age	Genotype	Mean±SEM	N° animals	Interactions	Normality test (Shapiro W.)	Variances (Levene)	Statistical test	Tukey post hoc test *p*-value	Signif.
Hot plate test (latency)										
46 °C	P21	WT	20.82±4.39 s	7	3-way ANOVA Log(data)	Failed	Passed	2-way ANOVA sqrt(data)	$p>0.05$	n.s.
		Clcn3$^{-/-}$	19.41±11.25 s	8						
	P60	WT	13.79±2.33 s	11					$p>0.05$	n.s.
		Clcn3$^{-/-}$	9.11±1.90 s	13						
48 °C	P21	WT	22.34±4.97 s	7		Failed	Passed	2-way ANOVA sqrt(data)	$p>0.05$	n.s.
		Clcn3$^{-/-}$	13.46±2.83 s	8						
	P60	WT	11.42±2.11 s	11					$p>0.05$	n.s.
		Clcn3$^{-/-}$	5.44±1.25 s	13						
50 °C	P21	WT	20.62±3.13 s	7		Failed	Passed	2-way ANOVA sqrt(data)	$p=0.045$	*
		Clcn3$^{-/-}$	11.19±2.19 s	8						
	P60	WT	15.71±2.11 s	11					$p<0.001$	***
		Clcn3$^{-/-}$	5.52±1.17 s	13						
52 °C	P21	WT	16.20±1.52 s	7		Failed	Passed	2-way ANOVA sqrt(data)	$p<0.001$	***
		Clcn3$^{-/-}$	5.90±1.18 s	8						
	P60	WT	7.44±0.62 s	11					$p=0.009$	**
		Clcn3$^{-/-}$	3.95±0.99 s	13						
Tail-flick test (latency)										
46 °C	P21	WT	11.90±2.16 s	8	3-way ANOVA sqrt(data)	Failed	Passed	2-way ANOVA sqrt(data)	$p=0.0013$	**
		Clcn3$^{-/-}$	3.18±1.28 s	9						
	P60	WT	19.38±2.46 s	8					$p=0.003$	**
		Clcn3$^{-/-}$	7.79±0.83 s	9						
48 °C	P21	WT	4.67±1.60 s	8		Failed	Passed	2-way ANOVA sqrt(data)	$p=0.02$	*
		Clcn3$^{-/-}$	1.66±0.29 s	9						
	P60	WT	9.09±0.66 s	8					$p=0.006$	**
		Clcn3$^{-/-}$	4.55±0.41 s	9						
50 °C	P21	WT	1.38±0.20 s	7		Failed	Passed	2-way ANOVA sqrt(data)	$p>0.05$	n.s.
		Clcn3$^{-/-}$	0.90±0.09 s	9						
	P60	WT	4.16±0.13 s	8					$p<0.001$	***
		Clcn3$^{-/-}$	2.13±0.19 s	9						

Supplementary Table S1. Acute thermal pain experiments in young P21 and adult P60 mice show increased thermal sensitivity and lower latencies in mutant than in WT animals. *$p< 0.05$, ** $p< 0.01$; *** $p< 0.001$; n.s. not significant; Signif. Significance.

Formalin test (flinches)										
Condition	Age	Genotype	Mean±SEM	N° animals	Interactions	Normality test (Shapiro W.)	Variances (Levene)	Statistical test	Tukey post hoc test *p*-value	Signif.
1 minute	P21	WT	34.38±4.88	8	3-way ANOVA sqrt(data)	Passed	Passed	2-way ANOVA	*p*>0.05	n.s.
		Clcn3$^{-/-}$	48.89±2.79	9						
	P60	WT	45.00±4.34	6					*p*>0.05	n.s.
		Clcn3$^{-/-}$	30.25±5.16	8						
5 minutes	P21	WT	3.88±1.84	8		Failed	Passed	2-way ANOVA sqrt(data)	*p*=0.02	*
		Clcn3$^{-/-}$	12.22±2.45	9						
	P60	WT	15.50±3.33	6					*p*>0.05	n.s.
		Clcn3$^{-/-}$	31.75±5.62	8						
10 minutes	P21	WT	1.00±0.46	8		Failed	Passed	2-way ANOVA sqrt(data)	*p*>0.05	n.s.
		Clcn3$^{-/-}$	6.44±1.16	9						
	P60	WT	9.83±1.96	6					*p*=0.016	*
		Clcn3$^{-/-}$	31.88±7.01	8						
15 minutes	P21	WT	2.63±1.03	8		Failed	Passed	2-way ANOVA sqrt(data)	*p*>0.05	n.s.
		Clcn3$^{-/-}$	9.67±4.44	9						
	P60	WT	20.83±4.50	6					*p*>0.05	n.s.
		Clcn3$^{-/-}$	41.12±8.55	8						
20 minutes	P21	WT	3.00±1.29	8		Failed	Passed	2-way ANOVA sqrt(data)	*p*=0.04	*
		Clcn3$^{-/-}$	8.44±1.24	9						
	P60	WT	24.67±4.39	6					*p*=0.007	**
		Clcn3$^{-/-}$	51.25±6.76	8						
25 minutes	P21	WT	5.63 ±1.64	8		Failed	Passed	2-way ANOVA sqrt(data)	*p*>0.05	n.s.
		Clcn3$^{-/-}$	8.78±1.08	9						
	P60	WT	16.50±3.89	6					*p*>0.05	n.s.
		Clcn3$^{-/-}$	35.50±7.77	8						
30 minutes	P21	WT	10.25±5.07	8		Failed	Passed	2-way ANOVA sqrt(data)	*p*>0.05	n.s.
		Clcn3$^{-/-}$	6.00±1.34	9						
	P60	WT	12.17±3.74	6					*p*>0.05	n.s.
		Clcn3$^{-/-}$	33.75±9.34	8						
35 minutes	P21	WT	5.13±1.99	8		Failed	Passed	2-way ANOVA	*p*>0.05	n.s.
		Clcn3$^{-/-}$	4.78±1.50	9						

	P60	WT	9.00±3.64	6				sqrt(data)	p>0.05	n.s.
		Clcn3$^{-/-}$	18.75±3.73	8						
40 minutes	P21	WT	1.00±0.50	8		Failed	Failed	Kruskall-Wallis ANOVA	p>0.05	n.s.
		Clcn3$^{-/-}$	2.11±0.81	9						
	P60	WT	2.33±1.96	6					p>0.05	n.s.
		Clcn3$^{-/-}$	14.00±5.51	8						

Supplementary Table S2. Inflammatory pain experiments in young P21 and adult P60 mice show an increased reaction in mutant than in WT animals. *p< 0.05, ** p< 0.01; n.s. not significant; Signif. Significance.
